# The limits of molecular signatures for pancreatic ductal adenocarcinoma subtyping

**DOI:** 10.1093/narcan/zcac030

**Published:** 2022-10-17

**Authors:** Manuela Lautizi, Jan Baumbach, Wilko Weichert, Katja Steiger, Markus List, Nicole Pfarr, Tim Kacprowski

**Affiliations:** Institute of Pathology, School of Medicine, Technical University of Munich, Munich, Germany; Chair of Experimental Bioinformatics, TUM School of Life Sciences, Technical University of Munich, Freising, Germany; Institute for Computational Systems Biology, University of Hamburg, Hamburg, Germany; Computational BioMedicine, University of Southern Denmark, Odense, Denmark; Institute of Pathology, School of Medicine, Technical University of Munich, Munich, Germany; German Cancer Consortium (DKTK), Partner Site Munich, Munich, Germany; Bavarian Cancer Consortium (BZKF), Munich, Germany; Institute of Pathology, School of Medicine, Technical University of Munich, Munich, Germany; Chair of Experimental Bioinformatics, TUM School of Life Sciences, Technical University of Munich, Freising, Germany; Institute of Pathology, School of Medicine, Technical University of Munich, Munich, Germany; Division Data Science in Biomedicine, Peter L. Reichertz Institute for Medical Informatics of Technische Universität Braunschweig and Hannover Medical School, Braunschweig, Germany; Braunschweig Integrated Centre of Systems Biology (BRICS), TU Braunschweig, Braunschweig, Germany

## Abstract

Molecular signatures have been suggested as biomarkers to classify pancreatic ductal adenocarcinoma (PDAC) into two, three, four or five subtypes. Since the robustness of existing signatures is controversial, we performed a systematic evaluation of four established signatures for PDAC stratification across nine publicly available datasets. Clustering revealed inconsistency of subtypes across independent datasets and in some cases a different number of PDAC subgroups than in the original study, casting doubt on the actual number of existing subtypes. Next, we built sixteen classification models to investigate the ability of the signatures for tumor subtype prediction. The overall classification performance ranged from ∼35% to ∼90% accuracy, suggesting instability of the signatures. Notably, permuted subtypes and random gene sets achieved very similar performance. Cellular decomposition and functional pathway enrichment analysis revealed strong tissue-specificity of the predicted classes. Our study highlights severe limitations and inconsistencies that can be attributed to technical biases in sample preparation and tumor purity, suggesting that PDAC molecular signatures do not generalize across datasets. How stromal heterogeneity and immune compartment interplay in the diverging development of PDAC is still unclear. Therefore, a more mechanistic or a cross-platform multi-omic approach seems necessary to extract more robust and clinically exploitable insights.

## Graphical Abstract

**Graphical Abstract:**
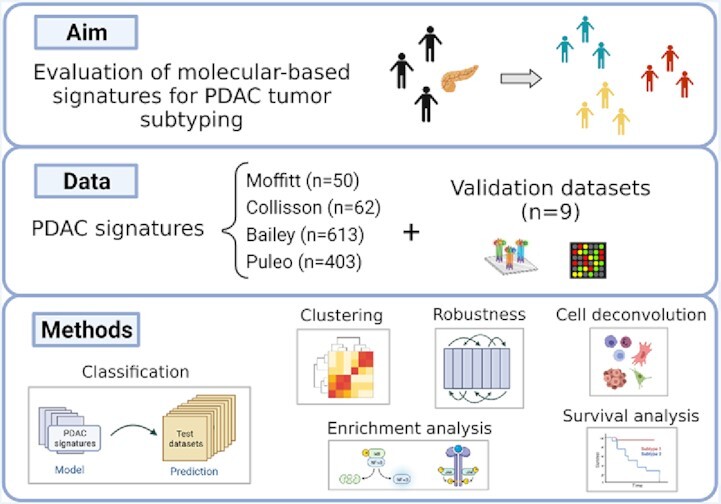
We evaluated the most widely used molecular signatures, revealing lack of reproducibility and robustness, and bias due to sample preparation, reinforcing the discussion about the challenging use of transcriptome data for PDAC subtyping.

## INTRODUCTION

### Background and context

Pancreatic ductal adenocarcinoma (PDAC) is the most frequent and aggressive malignant neoplasm of the pancreas. Its incidence has been increasing in the past decades and it is expected to rise further, reinforcing PDAC’s position as one of the deadliest cancer types and on the way of becoming the second leading cause of cancer-related death by 2030 (1). The absence of clear symptoms leads to a delayed diagnosis of the disease, where the tumor stage is often advanced and traditional treatments do not necessarily prolong the survival time. Around half of the patients show spread of distant metastases already at an early stage or when the tumor has still a small diameter (<2 cm), which renders surgical resection and radiotherapy no longer applicable (2,3). Despite progress in anti-cancer drug research, chemotherapy yields only minor survival advantages, which in combination with tumor resection reaches a five-year survival rate of 30% and above (4) while strongly affecting the quality of life due to its high toxicity (5).

More recently, immunotherapy has shown encouraging treatment results. However, the PDAC tumor microenvironment is widely heterogeneous and characterized by the presence of immunosuppressive cells which, in a dense stroma environment, makes the malignancy resistant to immunotherapy as well as chemotherapy (6–8).

Precision medicine, i.e. the stratification of patients into clinically actionable cancer subtypes based on the molecular characteristics of a tumor, is expected to significantly impact the outcome response (9). Different cancer subtypes present different mechanisms involved in carcinogenesis and are linked to different phenotypes (10). At the same time, patients within the same subgroup share common molecular patterns and genomic alterations, as well as similar clinical outcomes. Widely available transcriptomics data is particularly attractive for defining molecular subtypes as was initially demonstrated successfully in the context of breast cancer, where gene expression profiling drives subgroup classification (11,12). In the same way, researchers proposed the identification of molecular subtypes in PDAC.

### Molecular subtypes of pancreatic cancer

Many studies built up the current knowledge of PDAC molecular subtypes, proposing a tumor classification which reflects the biological and prognostic differences between patients. Here, we focus on the four transcriptomic and genomic studies most frequently discussed in literature for the stratification of PDAC into different groups. Definitions of molecular subtypes for pancreatic cancer were proposed by Collisson *et al.* (13) in 2011, Moffitt *et al.* (14) in 2015, Bailey *et al.* (15) in 2016, which are frequently considered as a gold standard for PDAC molecular subtyping, and Puleo *et al.* (16) in 2018, a more recent study.

Collisson *et al.* performed an unsupervised analysis on the combination of two microarray datasets, one coming from microdissected primary tumor samples (where the epithelium was devoid of the stroma) and a second one from whole tumor samples. The authors identify the signature of 62 genes for patients discrimination and proposed three subtypes: classical, quasi-mesenchymal (QM-PDA) and exocrine-like. To validate these subtypes, cell lines were tested for therapy response *in vitro*. The use of Gemcitabine and Erlotinib in human PDAC cell lines of known subtypes had different effects on the groups. Classical subtypes appeared to benefit more from Erlotinib, opposed to QM-PDA where Gemcitabine was more beneficial. In a validation of the 62 genes via unsupervised analysis on human and mouse cell lines, the Exocrine-like subtype was not observed. Furthermore, three additional publicly available expression datasets with corresponding survival data were used for subtype partitioning based on the 62 genes. Clustering these additional datasets together with their original data reproduced the three subtypes, whereas clustering the additional datasets individually lead to different clusters, suggesting that an independent reproduction of the subtypes in different datasets is challenging.

Moffitt *et al.* performed a virtual microdissection on the integration of multiple microarray data (primary and metastatic tumors, cell lines, normal pancreas and distant site adjacent normal samples). The transcripts were divided into categories linked with each input phenotype, and transcripts associated with tumor and stroma were used to define two distinct subtyping approaches. Tumor-specific subtypes rely on 50 tumor-related genes which stratify the patients into classical and basal-like subtypes, while 48 stroma-related genes were used to distinguish between normal and activated stroma subtypes. Survival analysis was used to assess how the subtypes differ prognostically. The discovered subtypes were further validated by sequencing of primary PDAC, patient derived xenografts, cell lines and cancer associated fibroblasts. While clustering on patient-derived xenografts supports the basal/classical classification, cell line models exhibit a prevalence of the basal-like subtype, implying cell lines are not suitable for such binary PDAC classification.

Bailey *et al.* used RNASeq data from pancreatic cancer samples of different histopathological subtypes that have >40% of tumor cellularity. *Via* clustering, they identified four clusters of subtypes: squamous, immunogenic, pancreatic progenitor and aberrantly differentiated exocrine (ADEX), which showed significantly different prognosis. To elucidate other biologically relevant characteristics of the subtypes, Bailey *et al.* integrated the transcriptomic analysis with a genomic analysis comprising whole-genome, deep-exome sequencing and gene copy number variation. A multiclass significance analysis of microarrays (SAM) analysis returned a list of 613 genes differentially expressed between the four subtypes. Bailey *et al.* employed a larger human cohort to assess the reproducibility of the classes, this time array-based and without assessing tumor cellularity. After submitting the mRNA expression profiles to the same clustering procedure, the authors report the discovery of the same tumor subclasses.

Puleo *et al.* collected microarray expression profiles of formalin-fixed paraffin-embedded samples from resected primary tumors. Clustering revealed five subtypes: pure classical, immune classical, desmoplastic, stroma activated and pure basal-like. The subtypes differ in immune and stromal composition as well as in tumor microenvironment, as shown by the authors through cellular and tumor compartment estimation performed with the use of transcriptomic tools and deconvolution of the data in independent components. Desmoplastic and immune classical are the subtypes with the highest immune infiltration, with the difference in desmoplastic which, in addition, shows elevated fibroblast and endothelial cells and inflammatory stromal features. As desmoplastic, also Activated Stroma presents high stromal content, specifically determined by the activated stromal components. Pure classical and pure basal-like tumors were found, respectively, well and poorly differentiated and both with low immune infiltration. Puleo *et al.* included a survival analysis of the proposed PDAC classes, finding subtypes with low immune infiltration (pure basal-like and activated stroma) associated with poor prognosis, as previously reported by Moffitt *et al.* The authors propose a signature of 403 genes able to cluster samples of independent datasets into the five subtypes. The validation of the subtypes was based on survival curves using three external public datasets, confirming the pure basal-like as the one with the worst outcome. The authors emphasize the comparison with the dual classification proposed by Moffitt *et al.*, the only study that takes the stroma compound into account, and conclude by addressing the attention to the possible existence of an intermediate group of samples with both basal and classical features.

Table 1 contains a summary of the main characteristics of each proposed subtype, divided by study.

**Table 1.** Overview on the PDAC molecular subtypes

**Bailey**	**Pancreatic progenitor**		**Squamous**			**ADEX**	**Immunogenic**
	Transcription factors PDX1, MNX1, HNFGS, FOXAS, HES1 related to early pancreatic development and associated with fatty acid oxidation, steroid hormone biosynthesis, drug metabolism and glycosylation of mucins		Overexpression of genes implicated in inflammation, hypoxia, metabolism, activated MYC pathway, TGF-β signaling, autophagy, cell proliferation. Activated α6β1, α6β4 and EGF signaling. Samples hypermethylated with consequent downregulation of endodermal cell fate genes causing loss of endodermal identity. TP53 mutation combined with upregulated TP63 expression linked to tumorigenesis and metastasis development			Transcriptional networks linked to later stages of pancreatic development and differentiation. Upregulated transcription factors (NR5A2, MIST1 and RBPJL) involved in acinar cell differentiation and regeneration after pancreatitis. Activated genes linked to endo crine differentiation and MODY diabetes, Exocrine secretion and regulation of beta cell development.	B and T cells infiltration. Expressed genes involved in antigen presentation and in B cell, CD4+ T cell, CD8+ T cell and Toll-like receptor signaling pathways. CTLA4 and PD1 upregulated and linked to immune suppression
	*Survival*: 23.7 months		*Survival*: 13.3 months			*Survival*: 25.6 months	*Survival*: 30 months
**Collisson**	**classical**		**Quasi-mesenchymal**			**Exocrine-like**	**-**
	High epithelial and cell adhesion-associated (GATA6) gene expression. KRAS mutation dependent.		Upregulation of mesenchyme associated genes			Upregulated Tumor digestive exocrine enzyme genes.	
	*Survival*: 23 months		*Survival*: 6.6 months			*Survival*: 19.7 months	
**Moffitt**	**Classical**		**Basal**			**-**	**-**
	Overexpressed adhesion-associated, ribosomal and epithelial genes (GATA6)		Overexpressed mesenchymal genes, also known to be upregulated in the basal subtype of breast and bladder cancer				
	*Survival*: 19 months		Survival: 11 months				
**Puleo**	**Pure classical**	**Immune classical**	**Pure basal-like**	**Desmoplastic**	**Stroma Activated**	-	-
	Low cellular infiltration. Enrichment of Gly12Arg KRAS mutation. Expression of hENT1. Low proteasome/apoptotic signal	Enrichment of Gly12Arg KRAS mutation. Expression of hENT1. High infiltration of immune cells (natural killer, B and T cells). Low proteasome/apoptotic signal.	Prevalence of CDKN2A or TP53 mutations	High expression of structural and vascularized stromal components Low tumor cellularity. High infiltration of immune cells, inflammatory components, fibroblast and endothelial cells.	High levels of stromal components (α-SMA, SPARC, FAP) and myofibroblast-like cancer-associated fibroblast		
	*Survival*: 43.1 months	*Survival*: 37.4 months	*Survival*: 10.3 months	*Survival*: 24.3 months	*Survival*: 20.2 months		

### State of the art for signatures validation and subtypes reproducibility

More than one study raised skepticism about the generalizability of the subtypes established by Collisson *et al.*, Moffitt *et al.* and Bailey *et al.* finding discrepancies in the biological relevance of the tumor classes, motivating us and others to reassess these findings through additional analyses (17–22). Subtypes proposed by Puleo *et al.* are not yet extensively evaluated by the literature, which gives us the incentive to include it and to offer a first critical assessment of their classifier.

Birnbaum *et al.* (17) assessed the prognostic value of Collisson *et al.*, Moffitt *et al.* and Bailey *et al.* classifiers by using a large cohort of primary tumor samples coming from 15 different public datasets. Their results independently confirmed the prognostic value of the Moffitt *et al.* and Bailey *et al.* gene classifiers set but not the Collisson *et al.* classifiers.

The Cancer Genome Atlas (TCGA) Research Network (18) performed the same unsupervised analysis as Collisson *et al.*, Moffitt *et al.* and Bailey *et al.* on their cohort of primary tumor samples, using each time the corresponding list of gene signatures. Once the clusters had been identified, they investigated their relationship with the purity of the tumor samples. They reported that subtypes such as immunogenic, ADEX (from Bailey *et al.*) and exocrine-like (from Collisson *et al.*) are related to low tumor purity, suggesting a contamination of the tumor samples from adjacent normal pancreatic tissue.

Rashid *et al.* (19) explored the ability of Collisson *et al.*, Moffitt *et al.* and Bailey *et al.* proposed gene set classifiers in prognostic differentiation and subtype replicability. They applied consensus clustering on nine independent patient cohorts for assessing clustering robustness and carried out survival analysis on those cohorts where survival information was available. They noticed that the two tumor-specific subtypes presented by Moffitt *et al.* are consistent across datasets and thus robust in reproducing different patient subgroups that are prognostically relevant.

Janky *et al.* (20), used the 62 genes proposed by Collisson *et al.* on a larger cohort of whole tumor samples. The three groups obtained from clustering the data showed an almost perfect overlap with the existing subtypes. However, the prognosis related to such clusters appears to be partially inconsistent with respect to the original study. Collisson *et al.* associated the Exocrine-like subtype with good prognosis, contrary to Janky *et al.* that found the subtype associated with bad prognosis, denoting inability of the signature to distinguish the subtypes according to the survival outcome assigned by the study.

### Inconsistencies of the subtypes

Collisson *et al.* themselves recognized that the Exocrine-like subtype might be the consequence of the presence of normal tissue exocrine cells in the sample. This was noticed while observing the subtypes including *in vitro* data, which were not linked to the exocrine-like subtype, confirmed by Moffitt *et al.* Similarly, several critiques have been directed at the ADEX subtype of Bailey *et al.*, which shows discordance in the subtype assignment and suggests a contamination of acinar cells (16,18).

The existence of the immunogenic subtype of Bailey *et al.* has also been subject to doubts. Maurer *et al.* (21) noticed that samples classified as immunogenic showed an enrichment in the stromal compartment, which might explain that immunogenic subtype originates from the tumor microenvironment. Sivakumar *et al.* (22) determined the functional pathways involved in each of the Bailey *et al.* subtypes and found a link between the immunogenic subtype and the cell cycle signaling pathway. Their samples appear to be low immunogenic. However, to verify that its discovery was not a direct consequence of dense immunological samples, its composition requires additional examination.

Puleo *et al.* estimated the cell population proportion of a cohort of resected primary tumors and found genes of acinar cells from healthy pancreas highly expressed in the ADEX subtype proposed by Bailey *et al.*, supporting the hypothesis that normal tissue contamination leads to a bias in these samples. This is further confirmed by assessing the survival curves of the four Bailey *et al.* subtypes, where ADEX does not show prognostic relevance.

Several studies reported subtypes driven by specific characteristics including tumor infiltration, stromal contribution and association with prognosis. Nevertheless, a coordination between such studies is missing. Only few of the proposed molecular subtypes can be matched, like the classical types from Moffitt *et al.*, Collisson *et al.* and Puleo *et al.* with the Pancreatic Progenitor from Bailey *etal.*. Beyond the disagreement in the number of subtypes and their nomenclature, the signature genes used to define the subtypes show poor overlap (Supplementary Figure S1).

In a binary classification we imply that two classes have opposing characteristics, e.g. samples enriched with basal or classical genes. However, several samples express signature genes belonging to both subtypes, showing hybrid features and hence making the classification difficult (18,23–25). Topham *et al.* (24) employed two logistic regression based tools designed for clinical application, one published by Moffitt *et al.* and one that was successively revised by Rashid *et al.* (19) (PurIST). A discrepancy was found for twelve percent of the samples that did not fit with the exclusive basal and classical subtype. The authors investigated the characteristics of such discordant samples, revealing intermediate patterns between the two classes and differences in survival. Discordant samples clustered together and showed a median expression level in between the two concordant classes. Inconsistencies in binary classification were also reported by Chan-Seng-Yue *et al.* (25), who found a cluster of tumors which inconsistently falls into either the two Moffitt *et al.* subtypes or the multi-classes schemas proposed by Collisson *et al.*, Bailey *et al.* and Puleo *et al.*. Single-cell RNA-Seq was executed to better interpret the tumor composition and revealed clusters of basal and classical cells proliferating in the same tumor, specifically in 13 out of 15 samples. Thanks to a single-cell level investigation, hybrid tumors at a bulk transcriptomic resolution can then be justified with the presence of cell populations that are usually attributed to a dichotomous classification. Such co-existence further suggests the interpretation of PDAC subtypes as a transcriptional continuum where hybrid subtypes can be attributed to the genomic succession from a classical-type to a basal-like type of tumor. Therefore, the heterogeneity can be linked to the tumor stage and the progression process (26,27).

The varying intratumor cell subpopulations exposed with single-cell transcriptomics, emphasize the idea of considering cancer subtyping as a spectrum of multiple molecular patterns and not as mutually exclusive molecular classes. The concept of a continuum signature was addressed by Nicolle *et al.* (28) who proposed a RNA-seq based pancreatic adenocarcinoma molecular gradient (PAMG). The gradient assigns a score to the tumors, and is able to grade them along a spectrum which is predictive of clinical outcome. The authors used patient-derived xenografts (PDX) samples, which were first assigned histologically to five subtypes, successively sequenced and subject to independent component analysis. The component most correlated with the histological classes constitutes the PAMG. Nicolle *et al.* further inspected the expression levels of classical and basal-like tumor types previously published and revealed a gradual shift between one subtype and the other, confirming their initial hypothesis of different molecular shades of PDAC not generalizable to a binary non-overlapping discrimination. PAMG was further compared to the binary classification performed by PurIST, showing a better performance in capturing heterogeneity and complexity of the samples.

### Problem statement

A key observation is that the molecular subtypes deduced by the previous studies are based on independent cohorts of patients with very diverse sample characteristics: Collisson *et al.* partially used laser microdissected samples, contrary to Moffitt *et al.* who microdissected the tumor from the stroma compartment virtually (*in silico*). Bailey *et al.* considered only samples with high epithelial content (>40%) whose histological subtype is not only ductal, while Collisson *et al.* and Moffitt *et al.* focused their attention on the ductal subtype alone. Puleo *et al.* used the full cohort without filtering samples for tumor cellularity extent or stromal compartment contribution. Data with different properties might induce inconsistent results in a comparison. Specifically, the different subtypes may simply reflect differences in the composition of the samples and hence the generalization and meaning of related biomarkers on different datasets remains unclear.

**Figure 1.**
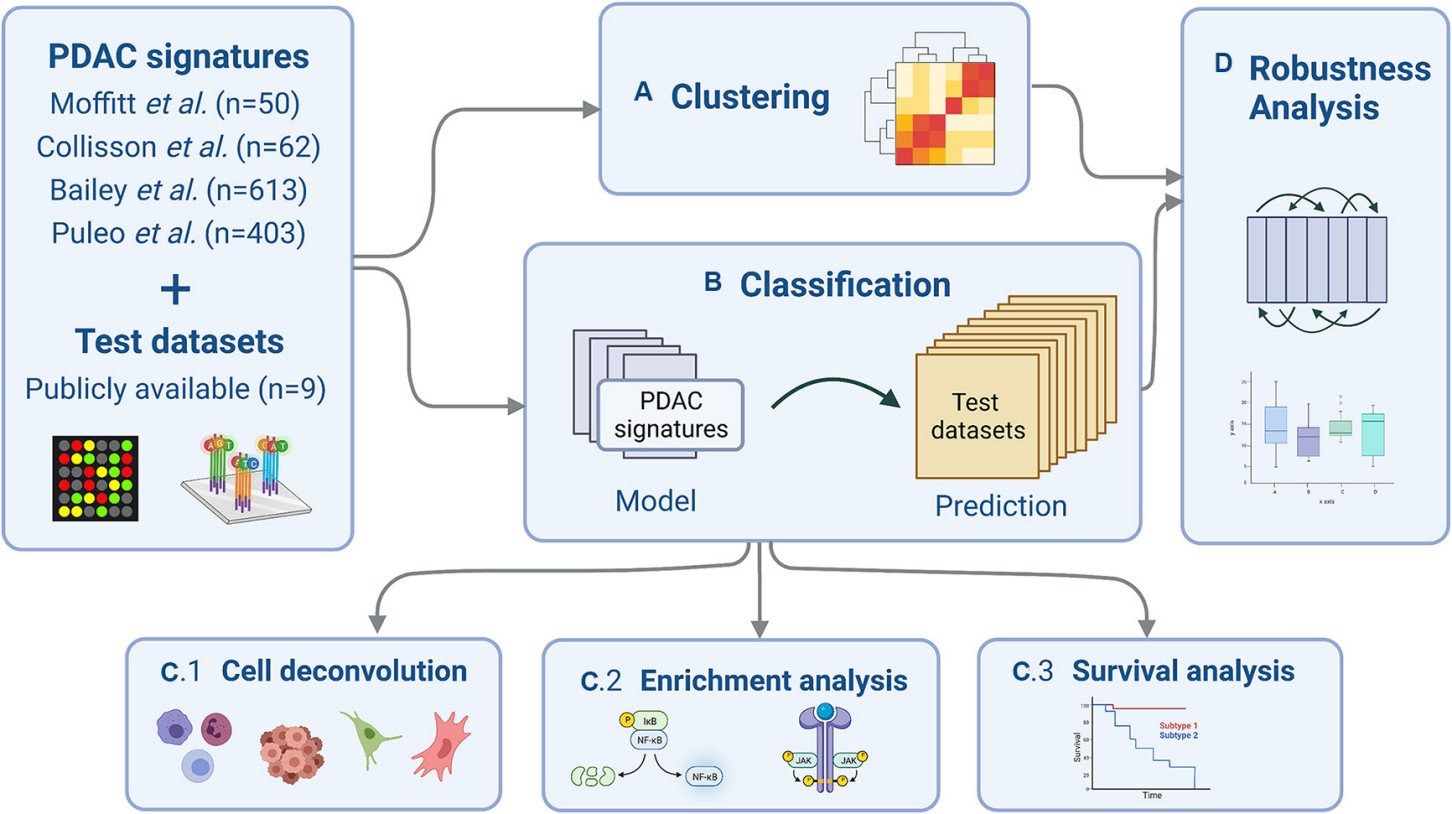
After collecting the PDAC signatures and the nine test datasets, we perform (**A**) clustering and (**B**) classification analysis. Based on the latter, we follow up with compositional analysis of the predicted subtypes *via* (**C.1**) *in silico* cell type deconvolution, (**C.2**) functional enrichment analysis and (**C.3**) survival analysis. (**D**) Finally, we also perform robustness analysis to learn if random signatures have comparable predictive power.

Most of the existing validation studies assess the prognostic power of the signatures by survival analysis on the patient clusters (17–19). Several studies investigated the composition of the public subtypes by observing the tumor compartment and the cellular environment of the samples (21,23,29,30). Nevertheless, a systematic study assessing the robustness of the signatures across different datasets is lacking. Here, we seek to answer the question if it is possible to replicate the same subgroups in a cohort that is not the one used for signature discovery. Moreover, it has been shown previously in breast cancer that random gene signatures often show performance comparable to published signatures (31). Hence, we also compare the performance of published signatures to randomly generated signatures of the same size (with respect to number of marker genes). In addition to survival analysis, which has been investigated before, we also link PDAC signatures to differences in cell type composition and compare them with respect to functional enrichment. With the latter, we reveal the mechanisms and processes of genes that represent the samples best, as shown by Xie *et al.* who investigated the immune functionalities linked to different risk groups of PDAC patients (32). Functional enrichment sheds light on the sample characteristics that the PDAC signatures represent. We demonstrate that they are only indirectly, if it all, linked to cancer subtypes. Workflow steps of the current study are illustrated in Figure 1.

## MATERIALS AND METHODS

### Distribution analysis

Gene expression distributions of the subtypes for each gene in the signatures are compared with a two-sided Wilcoxon rank sum test and Kruskal–Wallis test (Bonferroni corrected *P*-values < 0.05) when comparing two or more groups, respectively. A Wilcoxon test was further implemented pairwise, taking each gene at a time and comparing Puleo *et al.* subtypes, each one against the other four. *P*-values were corrected for multiple testing (Bonferroni, < 0.05). All tests were implemented in Python using the SciPy 1.6.0 library.

### Clustering

Agglomerative hierarchical clustering was implemented with the SciPy 1.6.0 Python library with correlation as metric. The analysis was applied to each dataset to identify signatures-related clusters, after filtering the gene expression matrix for signature genes. All data were *z*-score transformed before clustering. The number of examined clusters were two, three, four or five depending on the expected number of subtypes according to Moffitt *et al.*, Collisson *et al.*, Bailey *et al.* or Puleo *et al.*, respectively. We investigated the cluster differences using on each gene in the signature a two-sided Wilcoxon rank sum test for two and Kruskal–Wallis test for three, four or five clusters and taking the Bonferroni adjusted *P*-value (<0.05) distribution.

More details about the signatures overlapping with each datasets can be found in Supplementary Table S1.

Overlap percentage between clusters and real labels is computed using the Rand index (RI) and adjusted Rand index (ARI).

### Classification

Datasets used for classification were batch-corrected in order to avoid confounding between different sample preparation and origin. Correction was performed with the RemoveBatchEffect function included in limma 3.48.1 R package. Supplementary Figure S2 shows a principal component analysis of the nine datasets before and after correction. Only genes in common across all the dataset were considered for supervised analysis (more details in Supplementary Table S2). This filtering step might leave out genes belonging to the signatures that are not present in all the nine datasets. Furthermore, relevant genes which are highly expressed in the subtypes might be filtered out. On the other hand, considering only shared genes increases reliability and confidence of the results obtained from analyzing different datasets, along with allowing a fair comparison.

Data were *z*-score transformed before classification.

**Figure 2.**
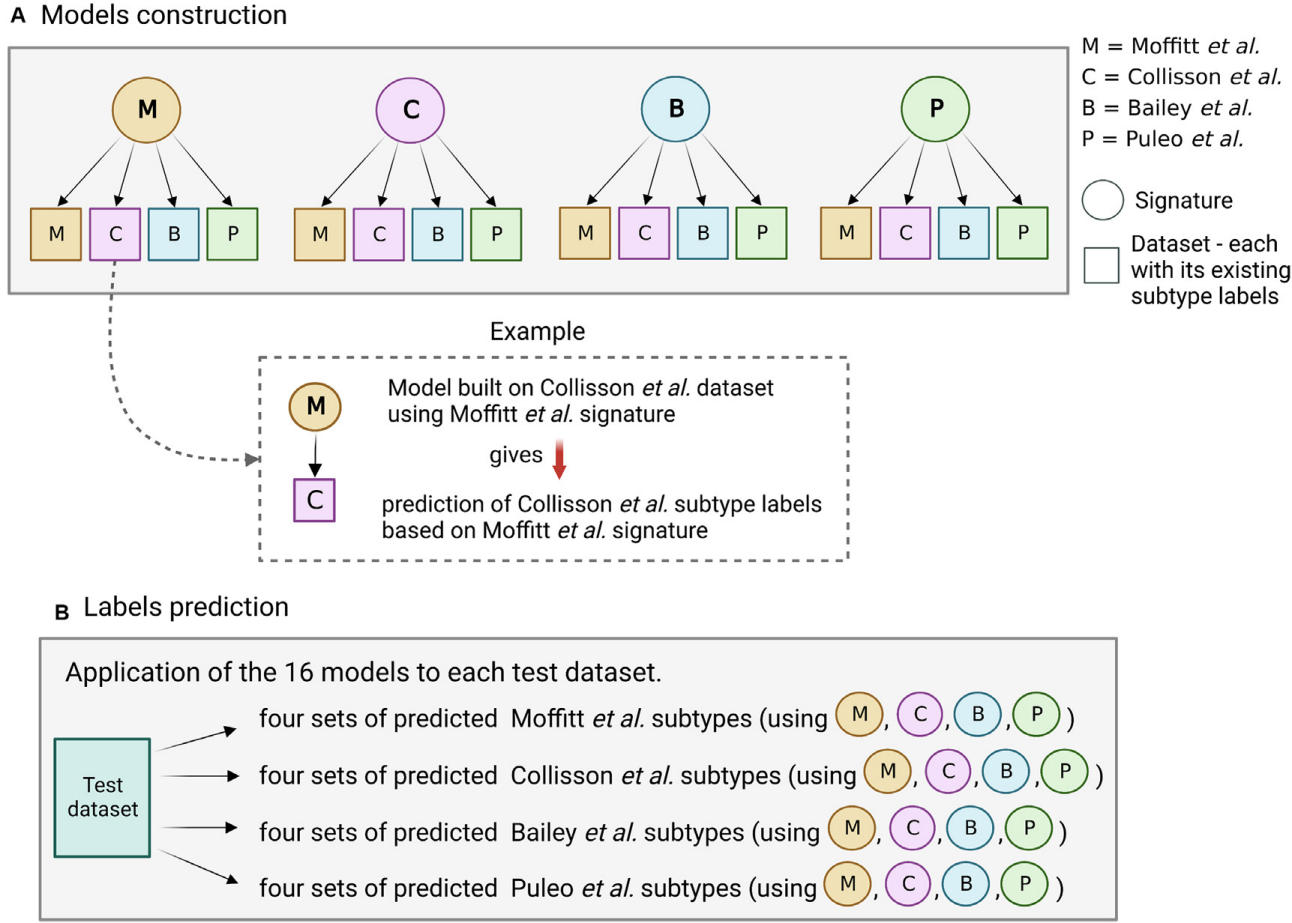
Illustration of the prediction models framework. (**A**) Models are trained using the four available signatures as a set of predictive features and changing, in turn, the training dataset, considering the one used by Moffitt *et al.*, Collisson *et al.*, Bailey *et al.* and Puleo *et al.* This results in sixteen models that are applied to nine test datasets. (**B**) Each model predicts the subtype labels linked to the training dataset: if we consider, for instance, a model trained on Moffitt *et al.* dataset, each test dataset will have four sets of predicted Moffitt *et al.* subtypes, obtained using the four signatures.

Classification was performed with the Random Forest Classifier in the Python package Scikit-learn 0.24.2, keeping the default values as parameters. Sixteen prediction models were built employing the four molecular signatures on the four datasets used for their discovery. Considering one dataset at a time, we filter the genes of each signature to employ them as predictive features. Samples in the current training dataset have available subtype labels which are used as target variables for the model. Each model is applied to a set of nine test datasets where samples are classified according to the target variable. The sixteen models are built with the aim of classifying different datasets into the four subtype schemes by using the four signatures. A graphic illustration of the model construction is shown in Figure 2. Average accuracy from 5-fold cross validation is used as an evaluation metric.

### Robustness analysis

Robustness of the signatures was assessed comparing their performance to random gene sets of the same size. Comparison is carried out using both clustering and classification. In classification, we run the models with four settings. First using as features the real signatures, then the random signatures and, lastly, shuffling the subtype labels while keeping the real signatures. Each setup was repeated 1000 times. Each average of the 5-fold cross-validation accuracy was stored and used for evaluation. We selected the signatures from every dataset and used them to generate clusters. Random signatures were used for the same purpose. The similarity between clusters obtained using the real signatures and random signatures are compared by computing the ARI. A pairwise ARI was used to compare clusters obtained from the random signatures. The analyses were performed in Python with Scikit-learn 0.24.2.

Strictly standardized mean difference (SSMD) (33) was used to assess the difference between ARI of the real signatures *versus* random signatures-derived clusters and the pairwise ARI computed on random signatures-derived clusters. SSMD was computed considering the two groups as independent and with unequal variance, with the following formula: $\beta = \frac{{{\mu _1} - {\mu _2}}}{{\sqrt {\sigma _1^2 + \sigma _2^2} }}$, where $\mu$ and ${\sigma ^2}$ stand for mean and standard deviation computed for group 1 and 2, respectively.

### Cell deconvolution and ssGSEA

We estimated the cellular microenvironment by deconvoluting bulk RNAseq data with the CIBERSORTx (34) method. Human pancreas single-cell transcriptome was obtained from Tosti *et al.* (35) and used to obtain a cell-type specific signature matrix for the deconvolution tool.

Single sample gene set enrichment analysis (ssGSEA) was performed in Python using the package GSEApy 0.10.5 (36–38). The repositories considered are KEGG (39), Reactome (40) and Gene Ontology terms (biological processes, molecular functions, cellular components) (41). ssGSEA can be understood as a gene-set level aggregated score that reflects a pathway or gene set's activity where genes in the set are up- or down-regulated in a coordinated, i.e. non-random fashion. Enrichment scores thus allow comparing pathway activity across samples and we use ANOVA and two-sided t-test to identify associated terms that reflect the phenotypic differences across the predicted subtypes from a functional pathways perspective (Bonferroni corrected *P*-values < 0.05). To find the terms significantly associated with each subtype, we look at the mean enrichment score of each term in each sample belonging to the same subtype. A term is considered associated with a subtype if it registers the highest mean enrichment score. We provide a summary in Figure 8 and Supplementary Figure S8 that show, for each enrichment library, the terms found significantly associated with the cohorts and with the different classes predicted by the sixteen classification models. Each figure shows the top 30 terms associated with the highest number of cohorts. For each subtype, we define a term upregulated or downregulated in that subtype by looking at the sign of the corresponding enrichment score.

### Survival analysis

Survival analysis was conducted with the Python package Lifelines 0.25.9 for datasets where survival information was available. Survival difference was assessed by computing pairwise log-rank tests between groups.

### PAMG evaluation

The PAMG scores were obtained *via* correspondence with the authors of the original publication (28). Four types of PAMG scores were derived from four different datasets (Patient-derived xenograft, Puleo *et al.* dataset, ICGC-RNAseq and ICGC-Array dataset) and we chose the score corresponding to the dataset, where possible, or ICGC-RNAseq and ICGC-Array for RNAseq and Array platform, respectively, in case the dataset was not in the PAMG options. Cox regression was performed in Python using the package Lifelines 0.25.9 and keeping the default parameters.

### Software and tools

Analyses were performed in Python 3.7.3 and R 4.1.0.

Scripts to reproduce analyses and figures are available on GitHub at https://github.com/biomedbigdata/PDAC-molecular-classifier-validation.

### Limitations of the study

We believe that differences in the platform used and normalization technique adopted by the different datasets might influence the signatures evaluation. Other variables like cohort size might influence the evaluation as well, since large cohorts are more suitable for such kinds of analysis. However, in order to limit the aforementioned potential confounding factors, we worked on *z*-scored values for the clustering-based evaluation and adjusted the datasets by taking different sources as a batch effect, after combining the data for classification analysis. In addition, to further control data composition differences, we decided to work only on human primary samples of ductal histological subtype.

## RESULTS

### Distribution analysis

**Figure 3.**
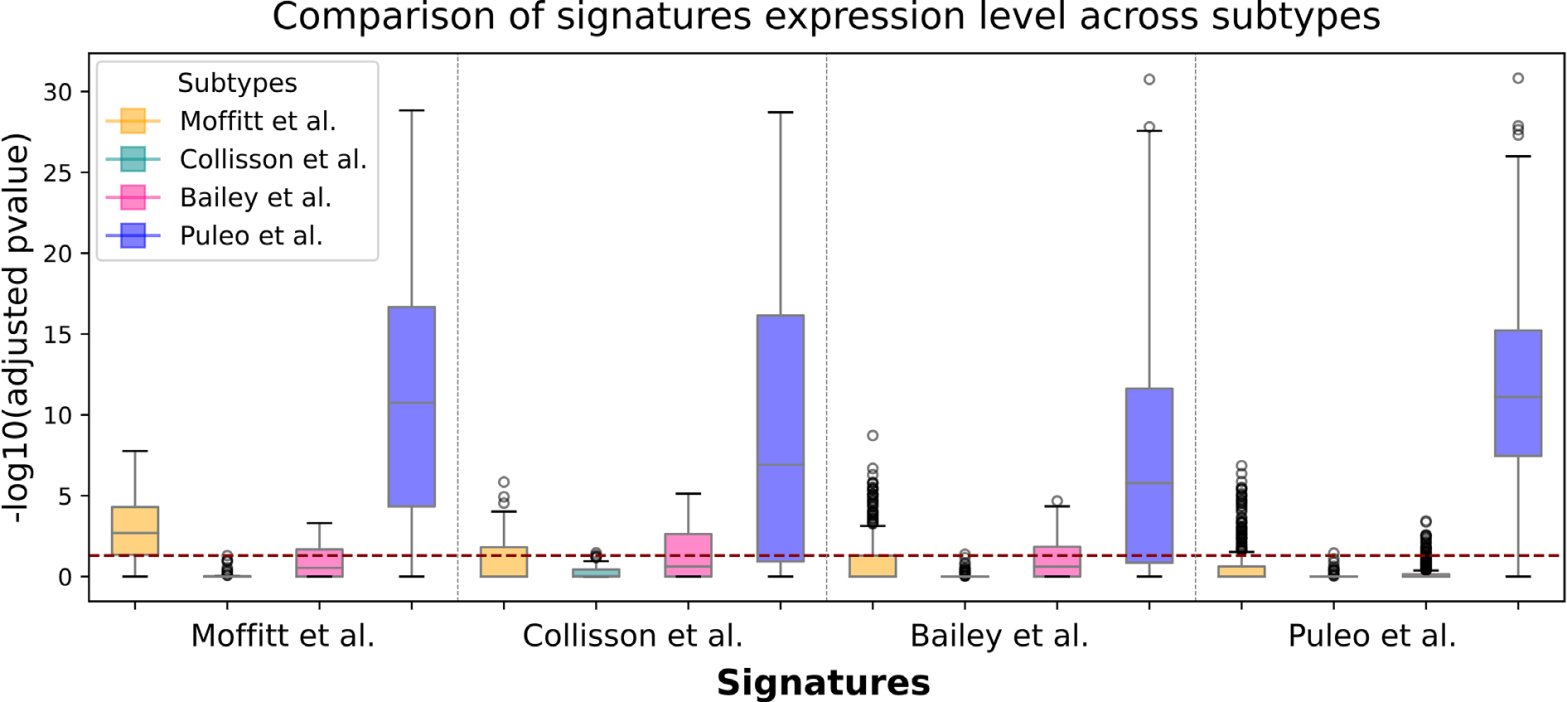
For each signature we assessed its variability across subtypes. Considering one gene at a time, we computed Wilcoxon rank sum test for two-class comparison and Kruskal–Wallis for more than two classes. The *P*-values returned by each test were adjusted with Bonferroni correction and were log-transformed. The horizontal dashed line indicates the threshold of significance (*P*-value = 0.05). The x-axis indicates the different signatures while the color indicates different subtypes.

We collected RNAseq and array expression profiles from nine datasets publicly available, including the data used by Moffitt *et al.*, Collisson *et al.*, Bailey *et al.* and Puleo *et al.* as discovery cohorts. For subtypes to be reliably discriminated, signature gene expression values should vary between tumor subgroups. As we hypothesize that signatures are rich in those genes whose expression across subtypes is significantly different, we tested for each individual gene its association with the subtypes (Wilcoxon rank sum test for two subtypes, Kruskal–Wallis test otherwise) (Figure 3). Interestingly, the five Puleo *et al.* subtypes are most clearly differentiated, regardless of the gene set considered for the test. Since the Kruskal–Wallis test assesses only if there is a different group, but does not identify it, we go further in detail to see which subtype is different from the others. We performed a pairwise comparison of each Puleo *et al.* subtype against the other four, finding the pure classical as the most distinguished, in some cases together with desmoplastic (Supplementary Figure S3). The subtypes from Collisson *et al.* have the most similar expression distribution irrespective of the signature used.

We further examined the consistency of the signatures across independent datasets in unsupervised and supervised analysis, followed by a comparison against random gene signatures and a functional evaluation of the subtypes.

### Clustering

Each of the nine datasets was subject to hierarchical clustering using only signature genes. We would expect that the resulting clusters reflect subtypes across different datasets. However, we observed that signature-based clusters accurately reflect subtypes only in their discovery dataset (Supplementary Figure S4A).

**Figure 4.**
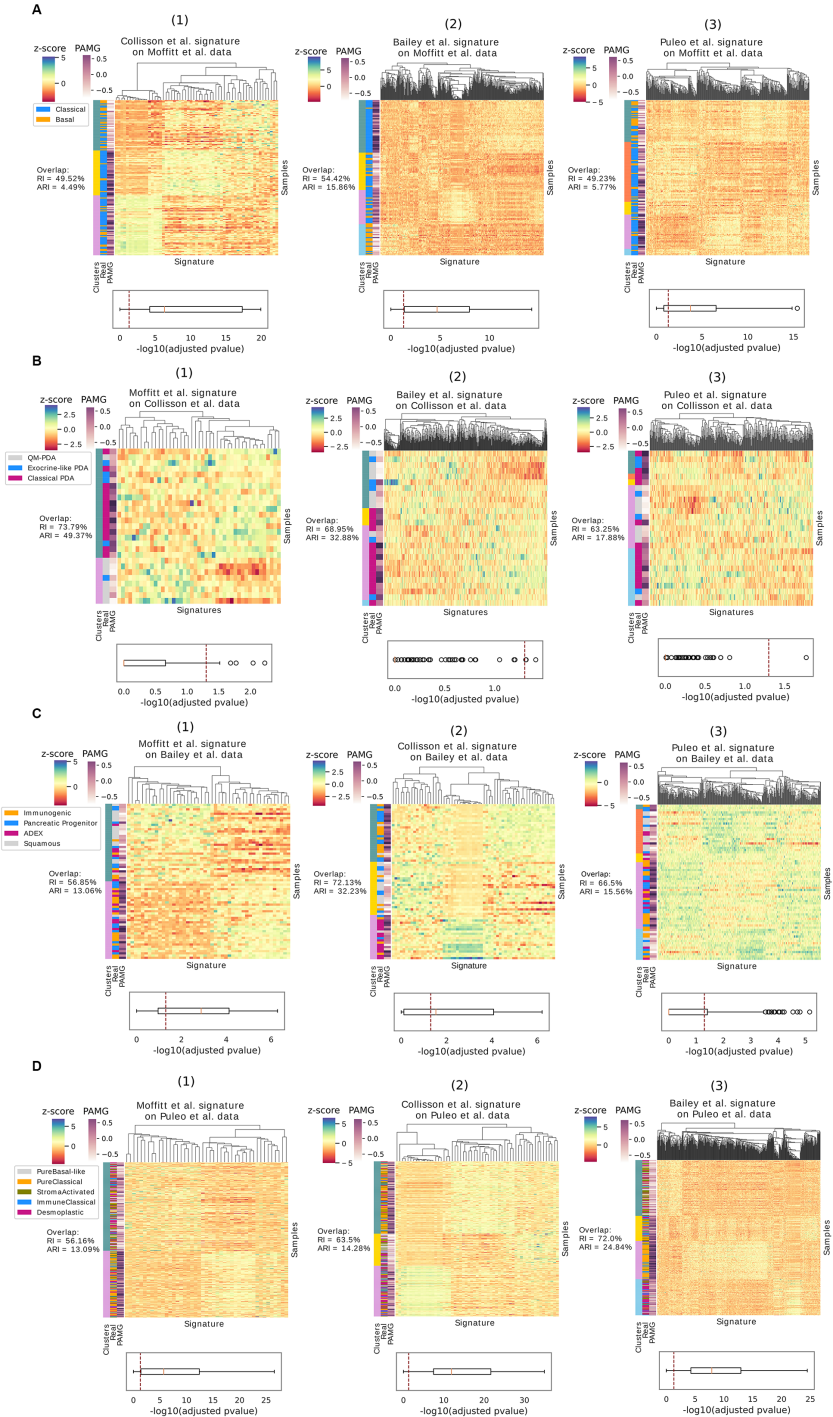
Hierarchical clustering of Moffitt *et al.* (**A**), Collisson *et al.* (**B**), Bailey *et al.* (**C**) and Puleo *et al.* (**D**) dataset, performed using Moffitt *et al.* (1), Collisson *et al.* (2), Bailey *et al.* (3) and Puleo *et al.* (4) signatures. Overlap between predicted clusters and real subtypes is observed on the left side of each heatmap, where samples are sorted by clusters. Overlap in percentage is expressed with RI and ARI. For each gene, association between signature expression profile and clusters is displayed with -log10(adjusted *P*-values) distribution below every heatmap, where *P*-values were obtained with Wilcoxon rank sum test for two and Kruskal–Wallis test for three, four and five clusters. Significance threshold of *P*-value = 0.05 is indicated with a vertical dashed line. PAMG values are visualized as additional color bar on the y axis of the heatmaps.

Although we found an overlap between some of the clusters and existing labels, we decided to quantify and assess the difference in expression between clusters. To do so, we used a Wilcoxon rank sum test for two and Kruskal–Wallis test for three, four and five clusters, and showed the adjusted *P*-value distribution (Figure 4 and Supplementary Figure S4, below every heatmap).

With the exception of the Collisson *et al.* and Bailey *et al.* signatures which cluster well on the data the authors used (Supplementary Figure S4A2 and A3), the expression profiles of the signature genes generally do not seem to discriminate well between clusters, as indicated by the –log_10_(adjusted *P*-value) distribution. The same outcome was found in Figure 4B1 with good RI but high similarity in expression levels between clusters. Nevertheless, we found cases of good overlap as well as good *P*-value distribution, as shown in Figure 4C2 and D3.

Clustering of other datasets is available in Supplementary Figure S4.

To further investigate the binary and multiclass subtypes, we compare the clusters with PAMG, the PDAC molecular gradient (28). While PAMG was established as a prognostic estimator, a poor and good prognosis can be linked to the aggressiveness of a subtype, like in the case of basal and classical which are the tumor types with worst and best prognosis, respectively (Table 1). We can see a good distinction especially in Figure 4A–C and Supplementary Figure S4, where PAMG, represented as a continuum, shows low values associated to basal-like (basal, quasi-mesenchymal and squamous) and higher values for classical-like subtypes (classical and classical PDA but not pancreatic progenitor in Figure 4C since signatures failed in clustering it). Good prediction was also found in Figure 4D if we reduce Puleo *et al.* subtypes to the two main categories of basal-like and classical-like subtypes (Table 1).

### Classification

A limitation when comparing subtypes across different studies is that the corresponding subtype labels are only available for the discovery cohort but not for the others. To enable further analysis and to assess the predictive potential of each signature, we build classification models that allow us to predict subtype labels for any of the four subtype schemes. Specifically, we use the Moffitt *et al.*, Collisson *et al.*, Bailey *et al.* and Puleo *et al.* datasets for training a random forest subtype classifier. For each of the different signatures, we build four models using the four datasets and their existing subtype labels, respectively, as illustrated in Figure 2. We use the sixteen models to classify each of the test datasets. When comparing predicted and original labels (for those datasets where PDAC molecular subtype labels were available), we observe that the real labels are mixed among the predicted labels (Supplementary Figure S5). The authors of the four studies declared a consensus across the subtyping strategies, where subtypes are supposed to overlap given their equivalent features, as also shown in Table 1. With this assumption, a sample classified as basal according to Moffitt *et al.*, should be predicted as Quasi-Mesenchymal when using a prediction model based on Collisson *et al.* subtypes, squamous when considering Bailey *et al.*, and pure basal, desmoplastic and stroma activated when considering Puleo *et al.* However, these subtypes were predicted to be both classical and basal, as shown in Figure S5a. Other examples can be found in Figure S5c, where the classical subtype is expected to be predicted only as pancreatic progenitor while what we see is samples being predicted inconsistently across multiple subtypes. This suggests that predictions are either not robust or that the different subtype schemes do not agree very well, as also suggested by the clustering analysis. As additional note, the Puleo *et al.* immune classical subtype was not predicted in Collisson *et al.* and Badea *et al.* data, independent of the signature that was used.

**Figure 5.**
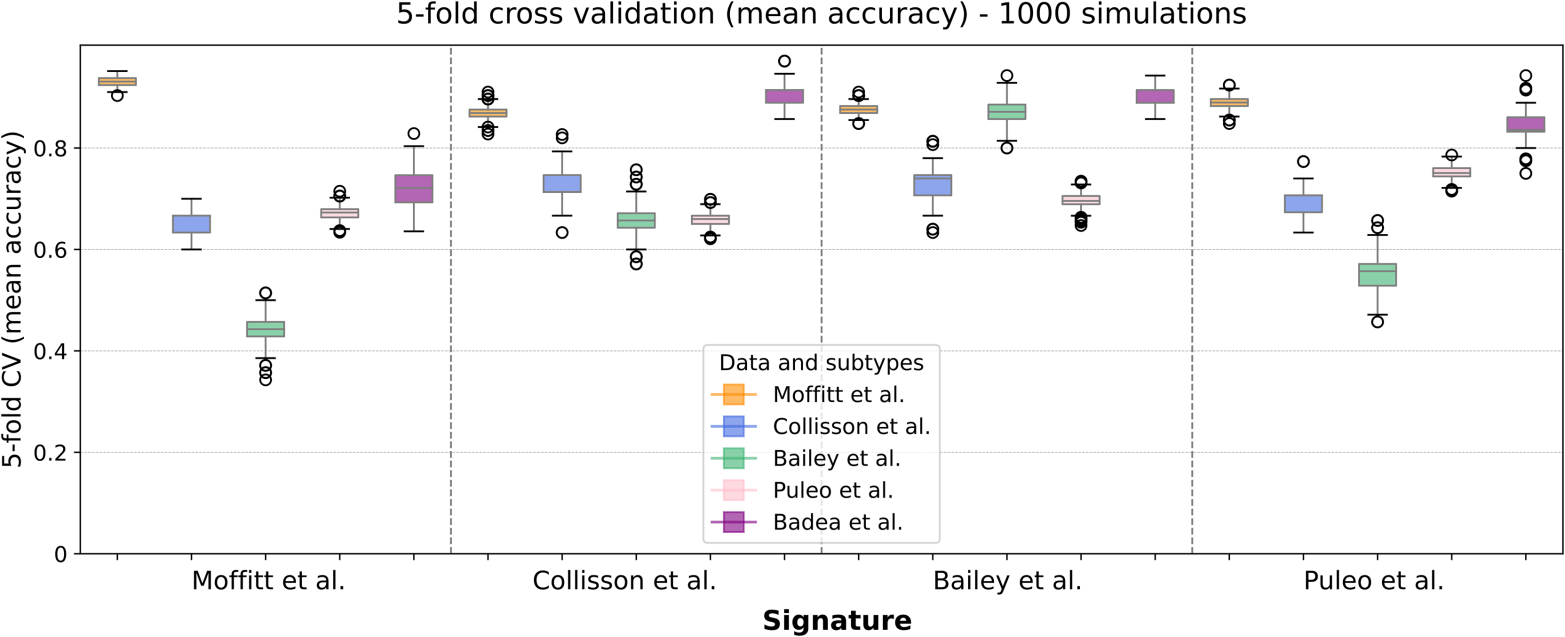
Distribution of the mean accuracy obtained by 1000 repeated 5-fold cross validation of classification. Use of signature (from left to right) from Moffitt *et al.*, Collisson *et al.* and Bailey *et al.* and Puleo *et al.* on the five datasets used for model training. Models built on Moffitt *et al.* as well as Puleo *et al.* mixed tissue dataset show better performance when compared with models built on exclusive tumor data.

To test if predictions are robust, we calculated the mean accuracy in a 1000 times repeated 5-fold cross-validation where, in addition to the Moffitt *et al.*, Collisson *et al.* Bailey *et al.* and Puleo *et al.* datasets, we also included the Badea *et al.* dataset, which comes with already assigned Collisson *et al.* tumor subtype labels. As shown in Figure 5, the Moffitt *et al.* signature showed the most dramatic differences in prediction accuracy. All the four signatures gave the best performance on the Moffitt *et al.* dataset for the prediction of their subtypes (basal and classical). Such high accuracy is likely owing to the simpler binary classification task. The Bailey *et al.* signature, with the largest number of genes, performs best across all datasets. The worst accuracy was registered with Moffitt *et al.* and Puleo *et al.* signatures when predicting Bailey *et al.* subtypes, the only two signatures which do not account for the exocrine signal.

### Robustness analysis

Several studies have shown that random gene signatures can outperform literature-reported signatures in outcome prediction (31,42), underlining that signature genes do not necessarily capture tumor biology. We hypothesize that random gene signatures may also be able to outperform published PDAC signatures in subtype prediction. To investigate this hypothesis, we took the models trained on the Moffitt *et al.*, Collisson *et al.*, Bailey *et al.* and Puleo *et al.* datasets and, for each dataset, compared the performance of a baseline model using signature genes against 1000 random gene sets of the same size as the corresponding signature. We additionally repeated model training after shuffling the subtype labels 1000 times. To assess if any of the models suffers from overfitting we computed 5-fold cross validation.

**Figure 6.**
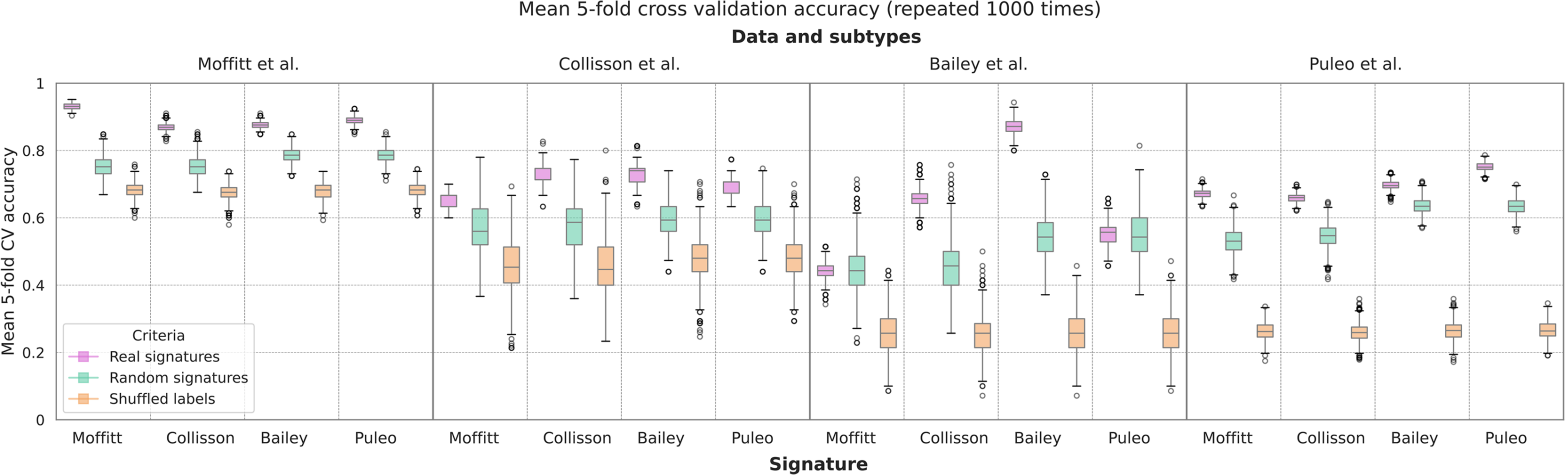
Evaluation of the classification ability of Moffitt *et al.*, Collisson *et al.*, Bailey *et al.* and Puleo *et al.* signatures used as features for classifying, in turn, their datasets. The same task was repeated using random signatures and shuffled labels of the target variable. Models are executed 1000 times with mean accuracy from 5-fold cross validation as evaluation metric.

As shown in Figure 6, some of the random signatures reach an accuracy similar to the literature-reported signatures, in some instances even outperforming them. The models based on the Moffitt *et al.* and Puleo *et al.* signatures to predict the subtypes from Bailey *et al.* yield a median accuracy equivalent to the one obtained using a signature of random genes. Moreover, the maximum accuracy registered in the 1000 runs is, for the signatures, significantly lower than the random ones (Table 2, (*) and (**)). Even though the median accuracy from real and random signature genes was comparable in only two cases, we found other two cases where the highest accuracy of random signatures observed among the 1000 runs was higher or as good as the actual signature.

**Table 2.** Highest accuracy registered by each model in 1000 runs. Models trained on (a) real signature genes, (b) random genes set of the same size as the signature, (c) real signatures but shuffled labels. Highest value among (a), (b) and (c) marked in bold. With (*), (**), (***) and (****) we point out the cases where the highest accuracy registered in the 1000 runs for using a random signature was higher or equal to the accuracy for using a real signature

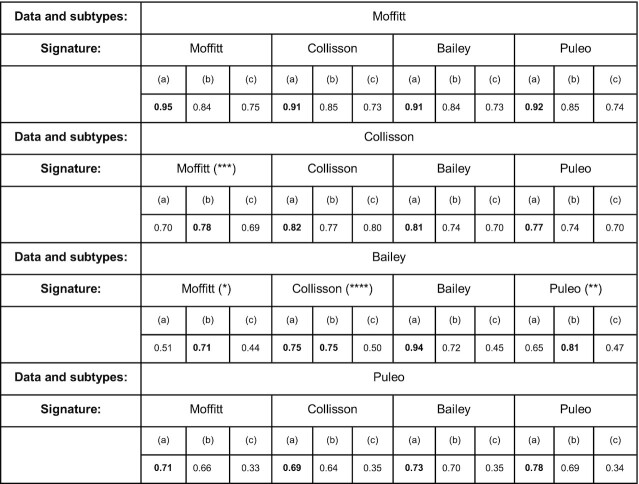

We observed this specifically with the Collisson *et al.* subtypes using the Moffitt *et al.* signature (Table 2, (***)) and with the Bailey *et al.* subtypes using the Collisson *et al.* signature where the highest accuracy for using a real signature was the same as using a random one (Table 2, (****)). Nevertheless, the prediction of subtypes other than Moffitt *et al.* show relatively poor classification performance. Beside some exceptions found with Collisson *et al.* signatures, models trained after label shuffling show an overall performance worse than models trained on real signatures, which is encouraging.

**Figure 7.**
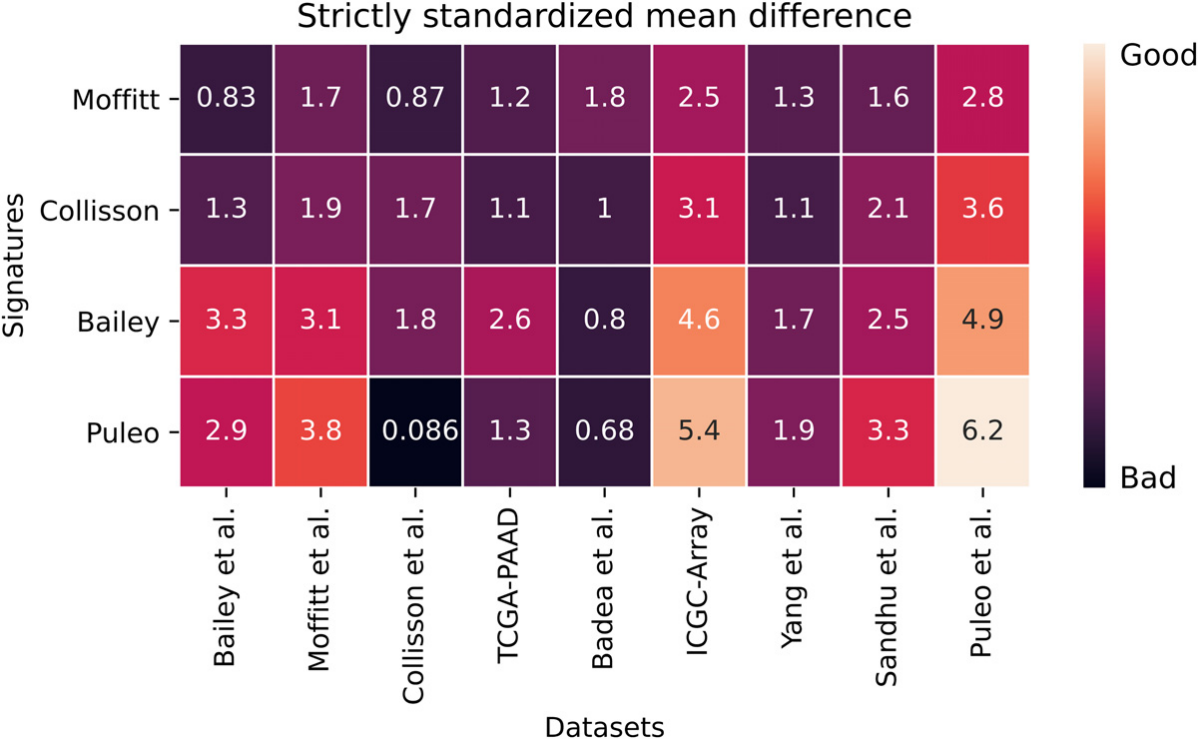
Strictly standardized mean difference (SSMD) comparing two distributions: ARI values of the overlap of clusters obtained with random signature with real signature-derived clusters are compared to ARI values obtained from pairwise comparison between only random signatures-derived clusters. Large values in the heatmap indicate that signature-derived clusters differ considerably from clusters of random signatures for a given dataset, i.e. the larger the value the better. The values shown are absolute values.

After assessing the robustness of the classification models, we focus on evaluating the clustering ability of the signatures when compared to clusters obtained from a set of random genes. To do so, we use the ARI to measure cluster similarity. We expect a low ARI when comparing clusters from random signature and real clusters, ideally much lower than the ARI we obtain when comparing pairwise random against random gene set clusters. Supplementary Figure S6 shows that across all nine cohorts most signatures achieve a very low ARI close to zero when compared against random gene set clusters. To summarize these findings, we computed the SSMD, which is a robust measure to quantify the difference of the means for the two distributions of ARIs values while accounting for the considerable variance (Figure 7). We notice differences for individual datasets which suggest that the dominant signal is not always related to the subtypes that the signatures aim to recapitulate. Puleo *et al.* signatures registered higher SSMD values with respect to the other three signatures, meaning that their gene panel generates clusters more different than the ones obtained by employing a random list of genes, compared to the other three signatures.

### Cell deconvolution and ssGSEA

With the trained classifiers (see Classification section), we were able to predict subtype labels, allowing us to investigate the functional characteristics of various subtype definitions across a broad set of datasets. We hypothesize that cellular composition of the samples has a major influence on the bulk transcriptome and that subtype definitions could reflect this heterogeneity rather than properties of the PDAC cells. To investigate if subtype definitions are confounded by stromal or immune cells, we used CIBERSORTx (43) to perform *in silico* cell type deconvolution. Specifically, we used a pancreas single-cell RNA-seq dataset to obtain cell-type-specific signatures allowing us to estimate stromal and immune cell enrichment scores (Supplementary Figure S7). As expected, Collisson *et al.* and Bailey *et al.*, which use rich tumor samples, are the datasets showing highest enrichment in ductal cells. Notably, we found a high abundance of pancreatic acinar cells in the Bailey *et al.* subtype ADEX and Exocrine-like from Collisson *et al.*, which can be considered as an effect of the presence of non-neoplastic tissue in the bulk samples. Classical subtypes, classical PDA from Collisson *et al.*, Pancreatic Progenitor from Bailey *et al.* and the pure classical from Puleo *et al.* where found, in some datasets, associated with ductal cells. The desmoplastic subtype was found enriched with macrophage cells, confirming the description from Puleo *et al.* and pointing towards immune infiltration. However, samples classified as desmoplastic were also found enriched with acinar cells. A macrophage infiltration was detected also in samples predicted as immunogenic with the use of Bailey *et al.* signatures, particularly observed in the datasets of Badea *et al.*, Yang *et al.* and ICGC-Array (Supplementary Figure S7). Overall, subtype schemes show comparable results, not dependent on the signatures but rather dependent on the dataset.

**Figure 8.**
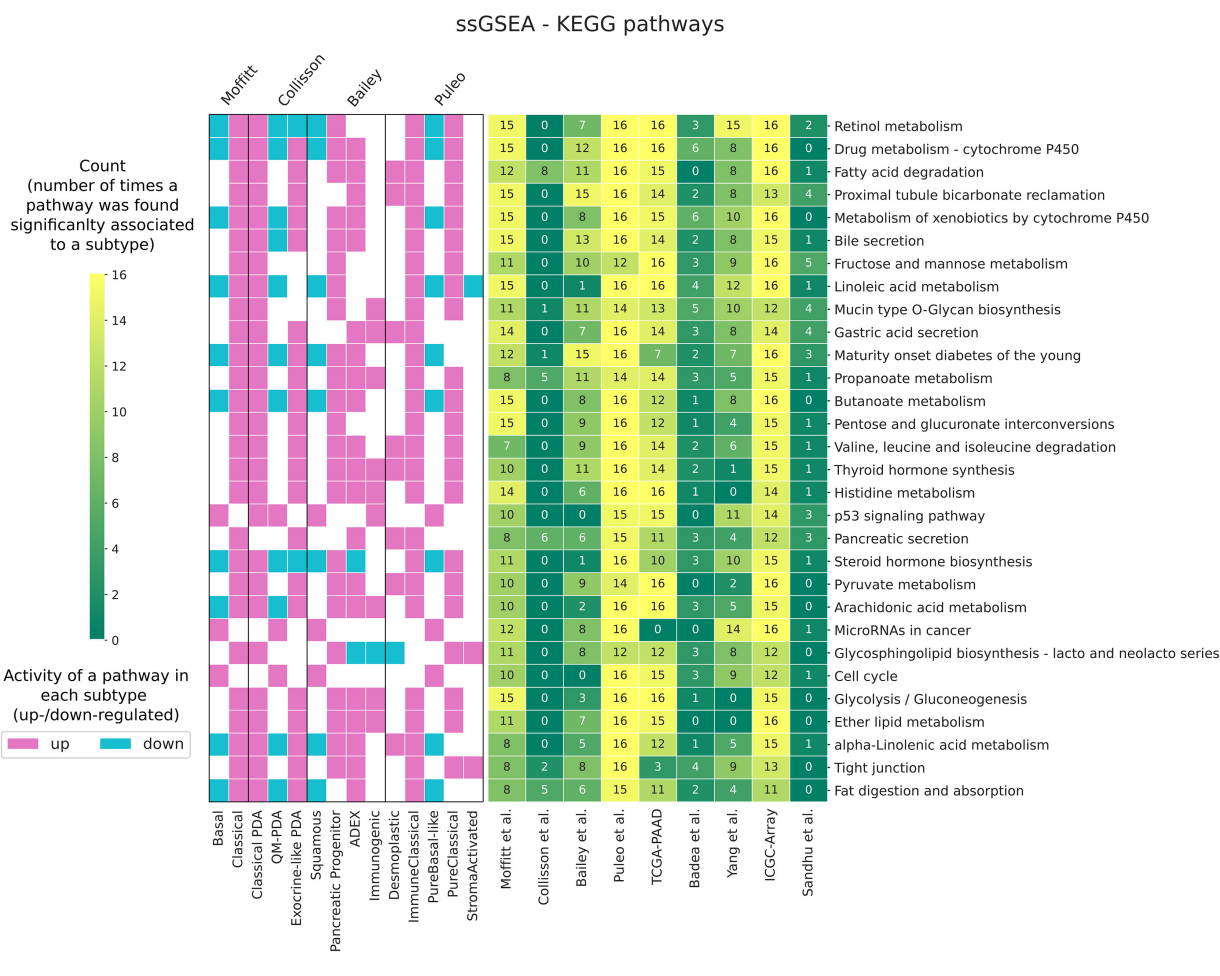
ssGSEA based on KEGG pathways. The table contains the number of times a term (on the rows) was found significantly associated with the subtypes comparison for each cohort (on the columns) using the subtypes predicted. Each term can be found associated up to 16 times considering the predictions executed using the 16 classification models. On the left, the vertical color bars show whether a pathway is up-/down-regulated and in which subtype. We found decreased metabolic activity in the basal subtype with respect to the classical, as shown by the left colorbar indicating down-regulation of metabolic pathways in the basal subtype.

Next, we performed single-sample Gene Set Enrichment Analysis (ssGSEA) to investigate which functional terms are associated with the different subtypes. For each of the sixteen classification models, we computed sample-wise enrichment scores across functional categories and tested them for significant association with the predicted subtype labels. Pathways we found significantly associated were mostly linked to metabolic activity, in particular fatty acid and lipid metabolism (Figure 8). Drug metabolism pathways like cytochrome P450 were associated with the Exocrine-like subtype, which is associated with drug resistance as also observed by Noll *et al.* (44). Other ssGSEA summary tables in Supplementary Figure S8.

### Survival analysis

**Figure 9.**
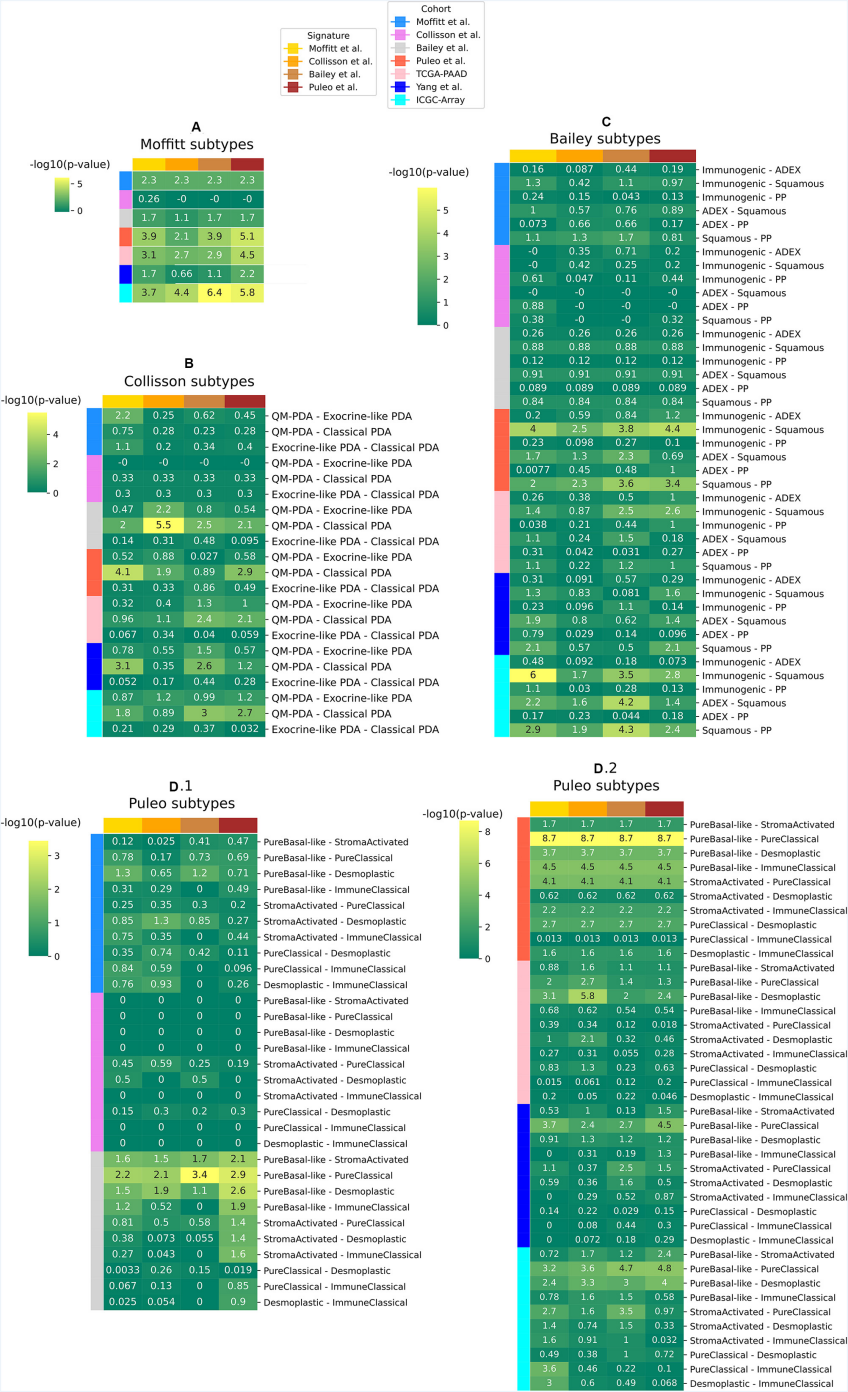
–log_10_(*P*-value) from a log-rank test computed comparing overall survival of the predicted subtypes for each dataset whose survival information was available (datasets on the rows). The four subtyping schema (A), (B) and (C) and (D) predicted using, in turn, Moffitt *et al.*, Collisson *et al.* Bailey *et al.* and Puleo *et al.* signatures. (**A**) Log-rank test computed between basal and classical subtypes from Moffitt *et al.*; (**B**) pairwise log-rank test between the three Collisson *et al.* subtypes; (**C**) pairwise log-rank test between the four Bailey *et al.* subtypes; (**D**) pairwise log-rank test between the five Puleo *et al.* subtypes.

In addition to insights into tumor biology, subtype definitions are used for determining clinically relevant differences in prognosis. We carried out a survival analysis comparing the overall survival of predicted subtypes across those datasets for which survival data was available. Figure 9 shows significant survival differences for the two subtypes proposed by Moffitt *et al.* (a), validating their original findings. For the Collisson *et al.* (b) and Bailey *et al.* (c) signatures, the pairwise *P*-values were only significant in few cases. Specifically, we observe highest –log_10_(*P*-value) when comparing survival curves of basal and classical subtypes: quasy-mesenchymal versus classical (Figure 9b) and pure basal-like versus pure classical (Figure 9 d.1 and d.2), beside the already binary classification from Moffitt *et al.*

## DISCUSSION AND CONCLUSION

In this study we evaluated the four major published molecular signatures for PDAC stratification. Despite the various sizes of subtype schemes proposed, direct comparisons are hampered by the differences of phenotypes. Yet, some overlap due to subtypes commonality should be expected (45).

Our findings indicate that these signatures appear inconsistent when applied to independent datasets, underlining their irreproducibility and further showing how the number and characteristics of PDAC subgroups still remain vague. Our results further suggest that current subtype definitions are mostly driven by tissue composition. In particular, the expression levels of the Bailey *et al.* signature frequently show low association with the four predicted clusters, suggesting that a three subtype schema is more plausible, supported by the hypothesis that the ADEX subtype is an artifact induced by the discovery data used and that can be attributed to contamination from healthy tissue (16,18,21,22). Bulk samples deconvoluted at a single-cell resolution revealed how samples enriched of pancreatic acinar cells were classified as ADEX as well as Exocrine-like, supporting the critique raised by other studies about the authenticity of the two mentioned subtypes (18,20). Our results present strong evidence that the expression profiles of these two PDAC subtypes along with their signatures are biased by the presence of adjacent normal pancreatic tissue, and suggest that samples with low tumor content were mistaken for an own subtype.

The signatures appeared to perform better than random genes, albeit the average classification accuracies are not too far apart, where even random genes achieve good accuracy. Bailey *et al.* signatures performed best in terms of accuracy when compared with the other two signatures along with random signatures or shuffled labels. In subtype classification, Moffitt *et al.* and Collisson *et al.* signatures (50 and 62 genes, respectively) show less predictive power than Bailey *et al.* and the Puleo *et al.* signatures (613 and 403 genes, respectively). The number of features used for a supervised analysis strongly influences the class prediction. Indeed, we observed a difference in accuracy between the smaller signatures, Moffitt *et al.* and Collisson *et al.*, and the larger ones, Bailey *et al.* and Puleo *et al.* with a plausible link to the amount of features employed for building the predictive model.

The signatures do not seem to determine prognostically relevant subtypes, as discovered through survival analysis. We found the comparison of all the basal-like against classical-like subtypes as the one linked to the most significantly different outcome, whichever is the signature implemented for classification. Therefore, we hope that future studies will consider the correction or removal of possible confounding factors, such as sample preparation, sampling bias and/or composition.

We detect the presence of an immune cell subpopulation, specifically macrophages, expressed in the immunogenic subtype from Bailey *et al.* and the desmoplastic from Puleo *et al.*. However, we also observed high expression of acinar cells in a portion of desmoplastic samples. Further focus should be dedicated to the understanding of the immune cell compartment. PDAC seems to generate a strong immune response since the initial state of the disease and it has been proved the impact of tumor-associated macrophages, regulatory T-cells and myeloid-derived suppressor cells on patient prognosis, denoting shorter survival given their prevalence in invasive cancer stages (46–48). In addition, studies focused on the examination of infiltrating immune-suppressive cells revealed distinct immune cells compartment in distinct tumor groups, suggesting the use of immune response as a strategy for the PDAC stratification (47,49). Additional assessment can confirm the potential existence of an immunogenic subtype and bring to light the prognostic role of immune mechanisms.

While the work of Moffitt *et al.*, Collisson *et al.*, Bailey *et al.* and Puleo *et al.* have advanced our understanding of molecular differences within cohorts of PDAC patients, we have to conclude that the proposed signatures are not well suited as a basis for clinical decisions without first accounting for differences in sample preparation and composition. However, the subtyping schema of Puleo *et al.* seems to be the one capturing most of the molecular heterogeneity, providing a broader spectrum for the understanding of PDAC differences. Indeed, the five subtypes they propose can be seen as subclasses of the two main classes basal and classical, the ones with the most robust differences in both composition and prognosis.

With our findings we highlight and confirm the existence of basal and classical, as more consistent and robust than a multi-class schema, as well as more prognostically relevant. Thus, a two tumor-specific classification seems more reliable and suitable for clinical application. Rashid *et al.* (19), with their PurIST classifier show robustness of the basal/classical discrimination in prognosis and therapy response, while the inclusion of additional known subtypes did not bring any relevant insight. PurIST was further used as a predictor for response to FOLFIRINOX and Gemcitabine, showing promising results. Despite the encouraging results in chemotherapy response estimation, PurIST lacks evaluation in clinical trials. Moreover, numerous studies faced challenges even from a binary classification (24,25,50).

From a broader point of view, the complexity of PDAC tumor microenvironment suggests the coexistence of features across the subtypes, stressing the focus on a continuous approach as the most suitable. The comparison between distinct classes from the four studies and the gradient stratification provided with PAMG gave promising results. Our findings confirm the ability of PAMG in representing samples classified as pure basal or pure classical-like, as well as intermediate non-dichotomous samples. On the other hand, the use of PAMG as a survival predictor showed poor performance when used on independent cohorts (Supplementary Table S5) suggesting that further investigation may be needed to make PAMG a robust prognostic marker across data sets. Thus, the use of transcriptome data for PDAC stratification still remains an open challenge.

The combination of multi-omics data (metabolomics, RNAseq, micro-RNAseq, whole-exome sequencing, SNP chip) for characterizing PDAC phenotypes was already discussed by Nicolle *et al.* (51), who found not only transcriptional changes but also alteration in methylation patterns and stromal compartment as important contributors to a binary classification into basal and classical. On the other hand, the genomic landscape including copy number aberrations was not found linked to phenotype or clinical outcome, stressing the focus on transcriptome, epigenome and stroma. Nicolle and colleagues observed in the tumor compartment of the basal samples a high expression of genes involved in glycolysis and cell cycle pathways. High deregulation of the Wnt signaling pathway was retrieved by mRNA genes and differentially methylated genes overexpressed in the basal subtype. The classical subtype was instead linked to normal pancreatic gastrointestinal cells pathways. The authors focused on the fundamental aspect to account for when investigating phenotypic differences, which combines epigenetic marks with gene expression. More recently, Cao *et al.* (52) carried out a study on the proteogenomic landscape (genomic, epigenomic, transcriptomic and proteomic) based on PDAC and normal samples. They address a detailed multi-omic analysis based on eight data types, aiming for the identification of biomarkers for early detection of PDAC in patients. Beside a multi-omic approach, we highlight the importance of an integrated analysis that implements them from a cross-platform point of view.

Several studies have shown that data at a single-cell resolution provides a more solid support for an unbiased definition of subtypes and investigation of the tumor microenvironment (25–27,53,54). Single-cell data thus constitutes a powerful tool to elucidate tumor progression such as the proliferation of immune cells and pancreatic stellate cells (54). Some studies have already shown how the integration of single-cell and spatial transcriptomic significantly contribute to further delineation of the PDAC tumor microenvironment (55–57). We believe that such innovative data can link neoplastic landscape and fibrotic stroma to the diverse malignancy states of the disease, and can help in the establishment of biomarkers to elucidate hybrid samples with mixed basal and classical features.

## DATA AVAILABILITY

Nine gene expression datasets were downloaded from public repositories.

Normalized microarray profiles from Moffitt *et al.* and Collisson *et al.* were downloaded from GEO under the accession codes GSE71729 and GSE17891, respectively. Only PDAC primary samples were kept.

Bailey *et al.* RNAseq profiles (ICGC PACA-AU) were obtained by author correspondence in a RSEM counts matrix that we normalized through variance stabilizing transformation in DESeq2. Non-ductal and non-primary histopathological subtype samples were excluded from this study.

Normalized Puleo *et al.* microarray expression matrix was downloaded from ArrayExpress (accession number E-MTAB-6134).

Normalized gene expression array data matrix from Badea *et al.*, Yang *et al.* and Sandhu *et al.* were downloaded from GEO (GSE15471, GSE62452 and GSE60980). Replicates in Badea *et al.* were dropped from this study.

ICGC Array data matrix was downloaded pre-processed from the International Cancer Genome Consortium (http://www.icgc.org) under the PACA-AU project and only primary samples of ductal histological type were kept.

Log2 of the counts for TCGA-PAAD RNAseq were obtained from Xena Browser (58). After filtering patients with PDAC primary tumor and ductal subtype, data were first converted from logarithmic to counts and then normalized with variance stabilizing transformation in DESeq2.

All probe ids were converted to gene ids, considering the median value if a gene name is mapped to multiple probes. An overview of the datasets used with respective gene and sample size can be found in Supplementary Table S3.

Molecular signatures and subtype calls assigned to the patients were downloaded from the corresponding publications of Moffitt *et al.*, Collisson *et al.*, Bailey *et al.* and Puleo *et al.* A list with the genes of every signature is given in Supplementary Table S4.

## Supplementary Material

zcac030_Supplemental_Files
