# Supplementary material for: The limits of molecular signatures for pancreatic ductal adenocarcinoma subtyping
Source: NAR Cancer. 2022 Oct 17;4(4):zcac030. doi: 10.1093/narcan/zcac030 (PMC9575186; doi:10.1093/narcan/zcac030)
Supplement: zcac030_Supplemental_Files [file zcac030_supplemental_files.zip › Revised_ Supplementary Figures.pdf]

Supplementary Figures

Figure S1

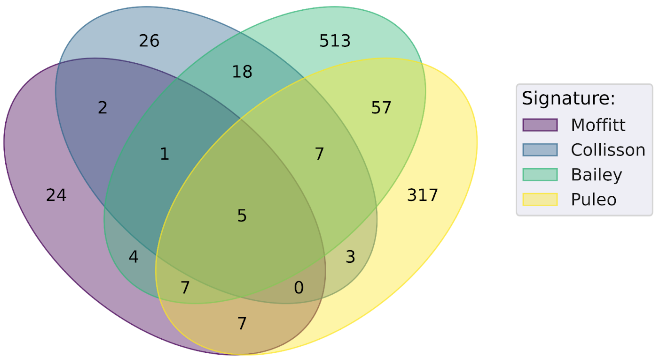

Figure S1. Venn diagram showing the number of overlapping genes across signatures.

Figure S2

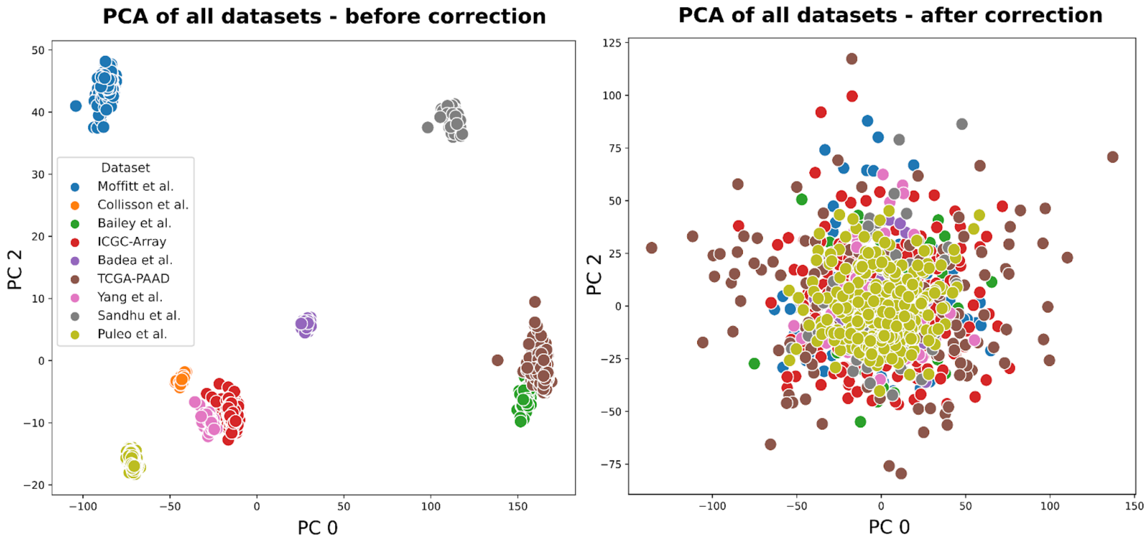

Figure S2. Principal Component Analysis (PCA) of the nine validation datasets combined. PCA before correcting the data for different sources of cohorts as a confounding factor (left). The right plot shows the sample of each cohort after the source correction.

Figure S3

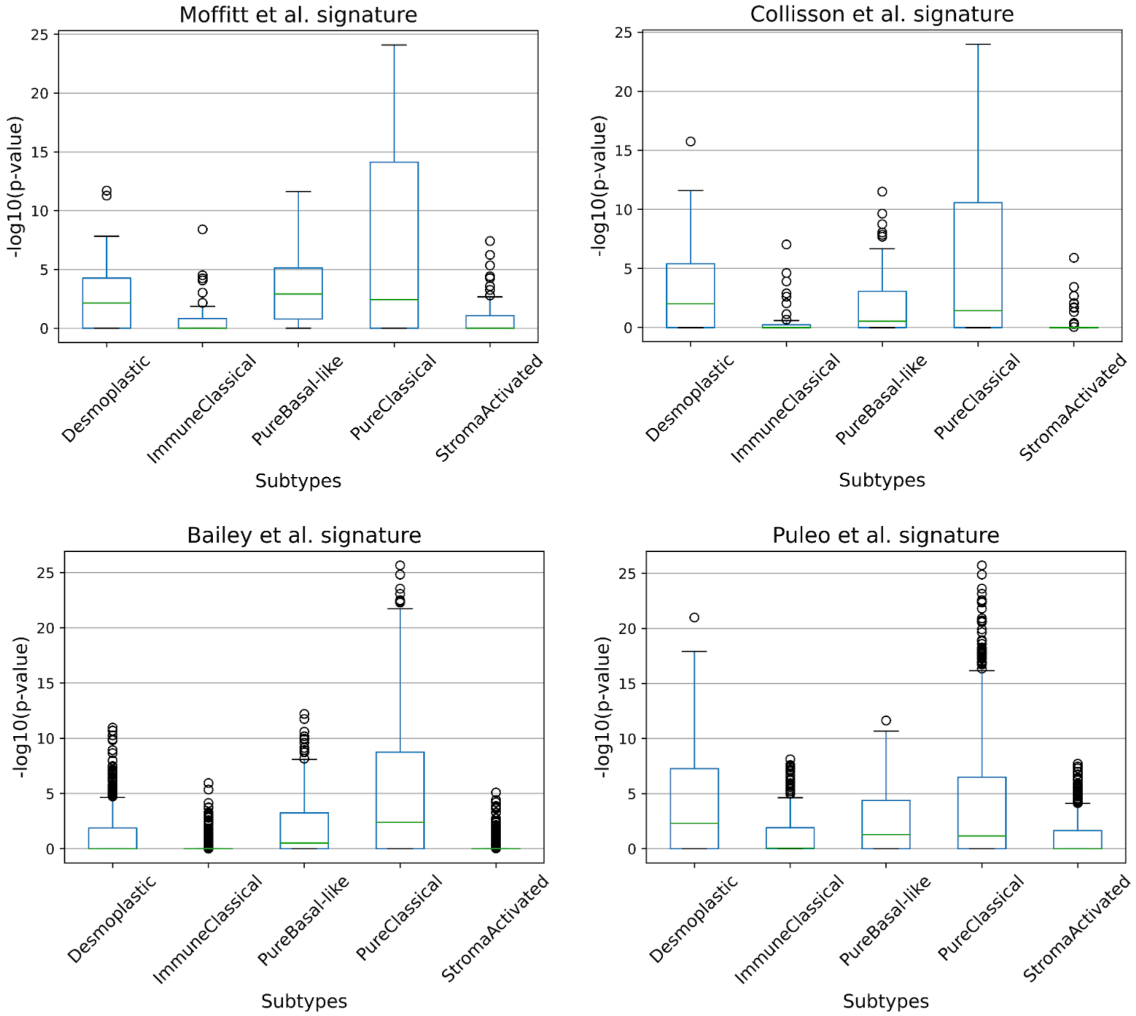

Figure S3. Pairwise Wilcoxon test on Puleo *et al.* subtypes, performed comparing one subtype *versus* all the others. Tests were carried out using one gene at a time from the four signatures. For each subtype, compared against the remaining four, we show the distribution of -log10 of the p-values corrected for multiple testing.

Figure S4

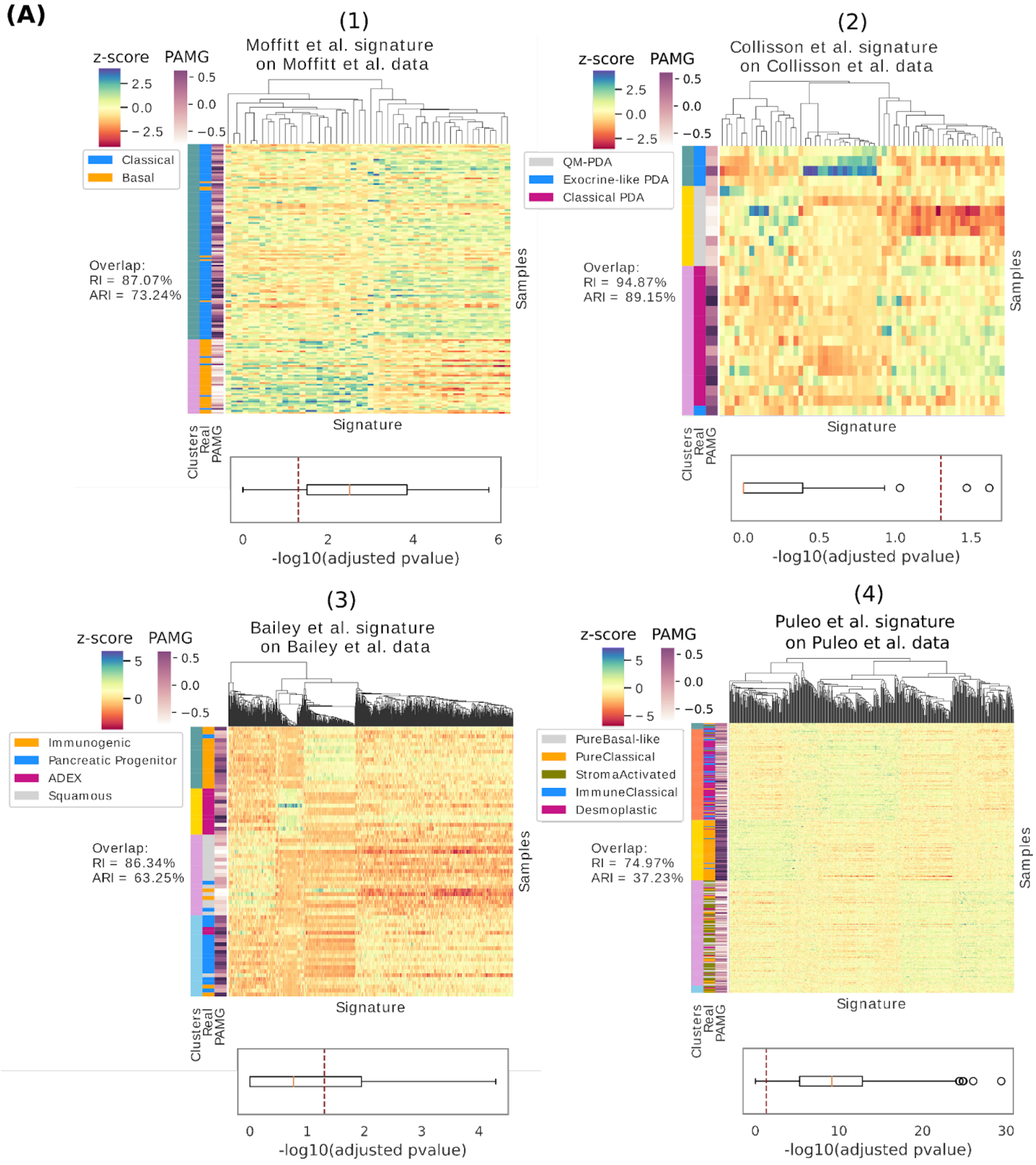
(A)
(1)
Moffitt et al. signature
on Moffitt et al. data
z-score
PAMG
Classical
Basal
Overlap:
RI = 87.07%
ARI = 73.24%
Samples
Signature
Clusters
Real
PAMG
-log10(adjusted pvalue)
(2)
Collisson et al. signature
on Collisson et al. data
QM-PDA
Exocrine-like PDA
Classical PDA
Overlap:
RI = 94.87%
ARI = 89.15%
(3)
Bailey et al. signature
on Bailey et al. data
Immunogenic
Pancreatic Progenitor
ADEX
Squamous
Overlap:
RI = 86.34%
ARI = 63.25%
(4)
Puleo et al. signature
on Puleo et al. data
PureBasal-like
PureClassical
StromaActivated
ImmuneClassical
Desmoplastic
Overlap:
RI = 74.97%
ARI = 37.23%

(B)

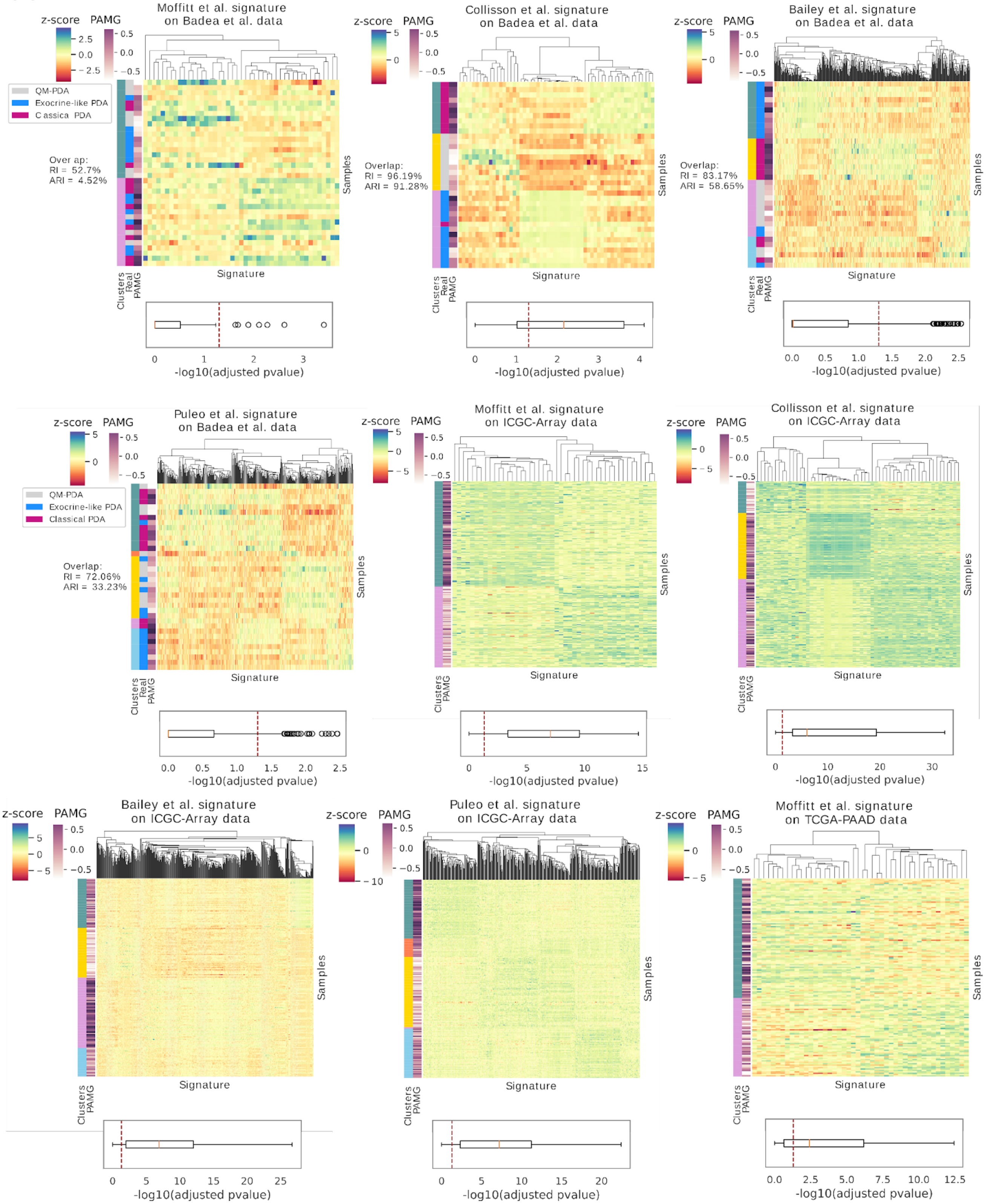
Moffitt et al. signature
on Badea et al. data
z-score
PAMG
QM-PDA
Exocrine-like PDA
Classical PDA
Overlap:
RI = 52.7%
ARI = 4.52%
Samples
Signature
Clusters
Real
PAMG
-log10(adjusted pvalue)
Collisson et al. signature
on Badea et al. data
Overlap:
RI = 96.19%
ARI = 91.28%
Bailey et al. signature
on Badea et al. data
Overlap:
RI = 83.17%
ARI = 58.65%
Puleo et al. signature
on Badea et al. data
Overlap:
RI = 72.06%
ARI = 33.23%
Moffitt et al. signature
on ICGC-Array data
Collisson et al. signature
on ICGC-Array data
Bailey et al. signature
on ICGC-Array data
Puleo et al. signature
on ICGC-Array data
Moffitt et al. signature
on TCGA-PAAD data

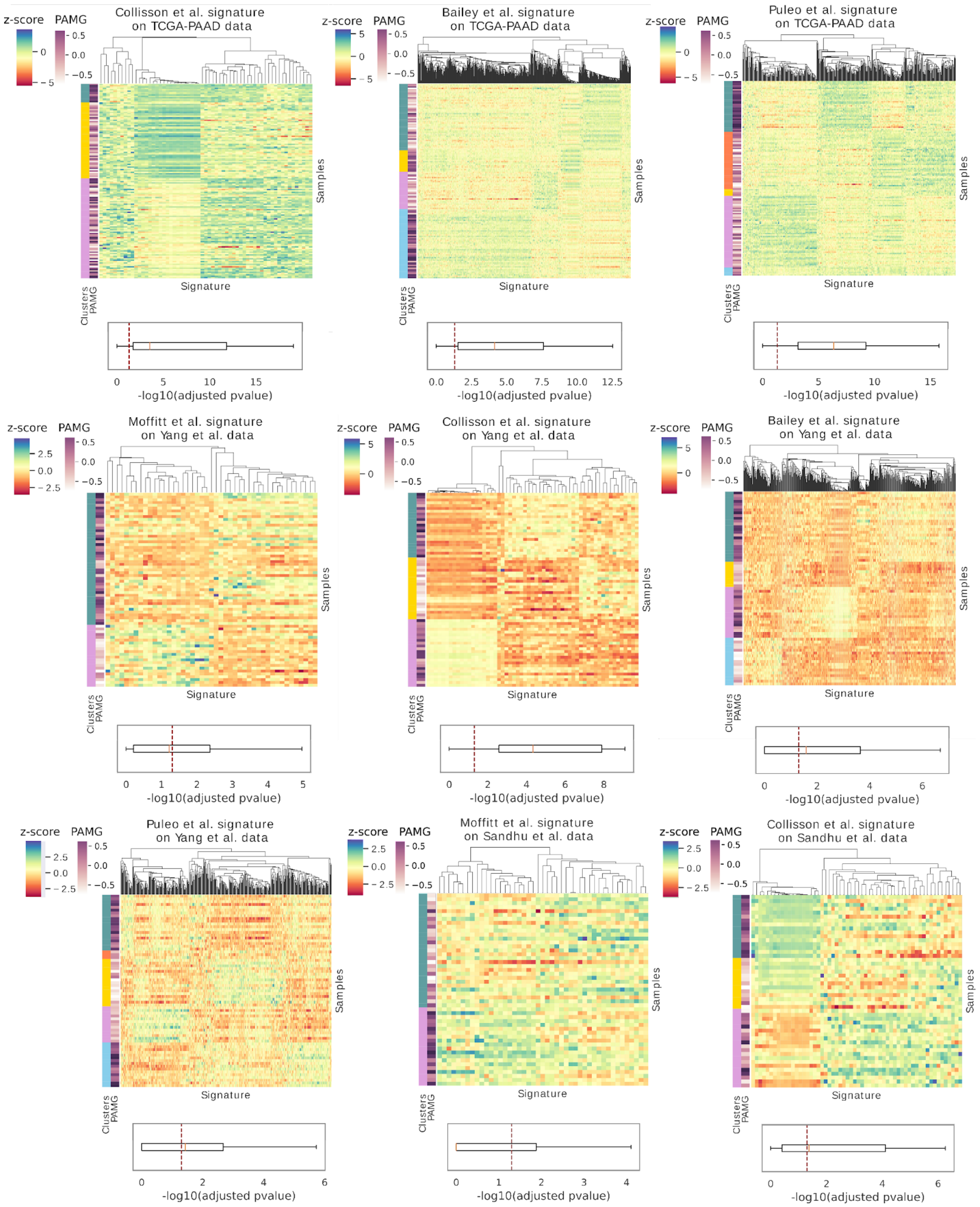
Collisson et al. signature on TCGA-PAAD data
Bailey et al. signature on TCGA-PAAD data
Puleo et al. signature on TCGA-PAAD data
Moffitt et al. signature on Yang et al. data
Collisson et al. signature on Yang et al. data
Bailey et al. signature on Yang et al. data
Puleo et al. signature on Yang et al. data
Moffitt et al. signature on Sandhu et al. data
Collisson et al. signature on Sandhu et al. data
z-score
PAMG
Samples
Signature
Clusters
PAMG
-log10(adjusted pvalue)

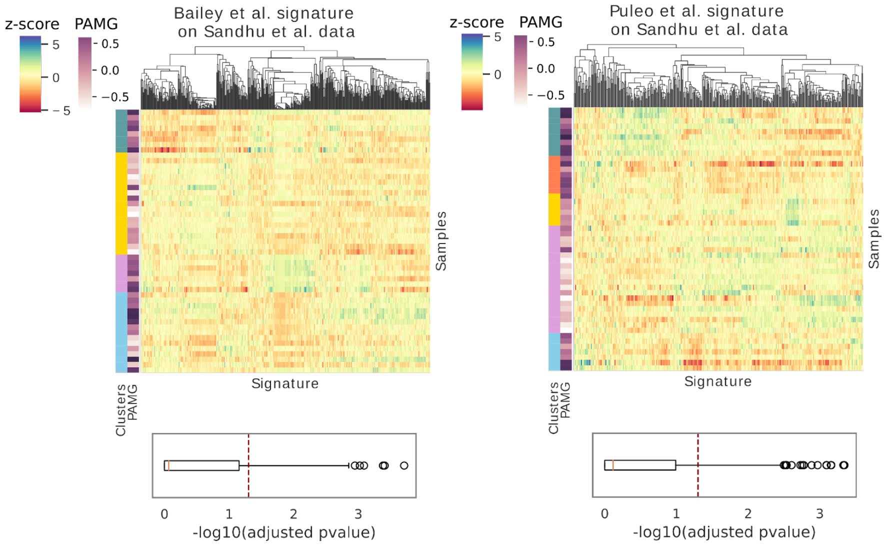

Figure S4. Hierarchical clustering using the signatures from Moffitt *et al.*, Collisson *et al.*, Bailey *et al.* and Puleo *et al.*. (A) Signatures applied on the same dataset used for their discovery. (B) Signatures applied to the Badea *et al.*, ICGC-Array, Sandhu *et al.*, TCGA-PAAD, Yang *et al.* z-scored datasets. Below every heatmap, a boxplot shows the -log10(adjusted p-values) distribution obtained assessing the difference in expression between clusters computing, on each gene in the signature, Wilcoxon rank sum test for two and Kruskal-Wallis test for three, four and five clusters. A vertical dashed line indicates the significance threshold of p-value=0.05. (*) Clusters of Badea *et al.* are compared with the real subtypes assigned in the study of Collisson *et al.*.

Figure S5

(a) Prediction of Moffitt *et al.* labels

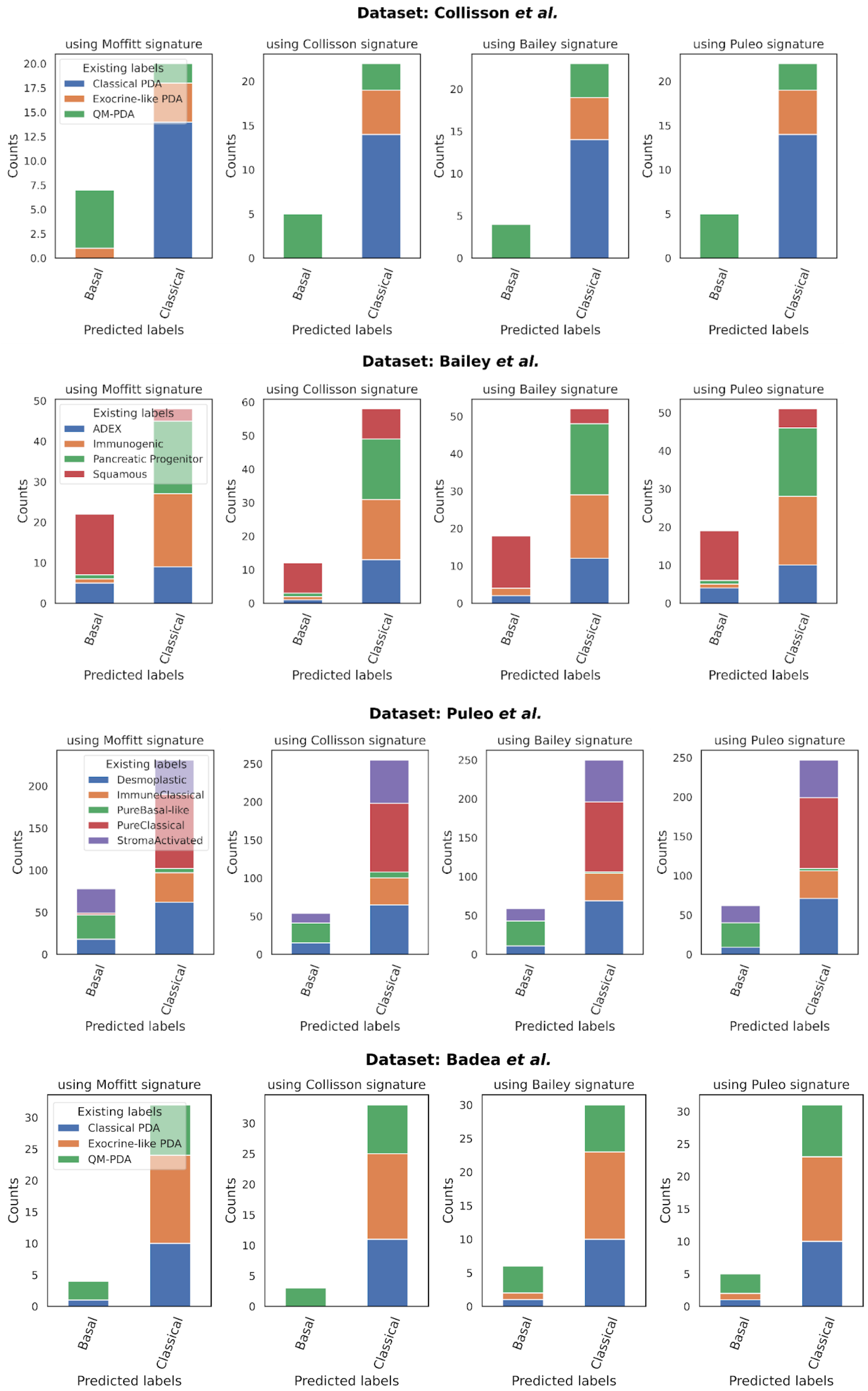

(b) Prediction of Collisson *et al.* labels

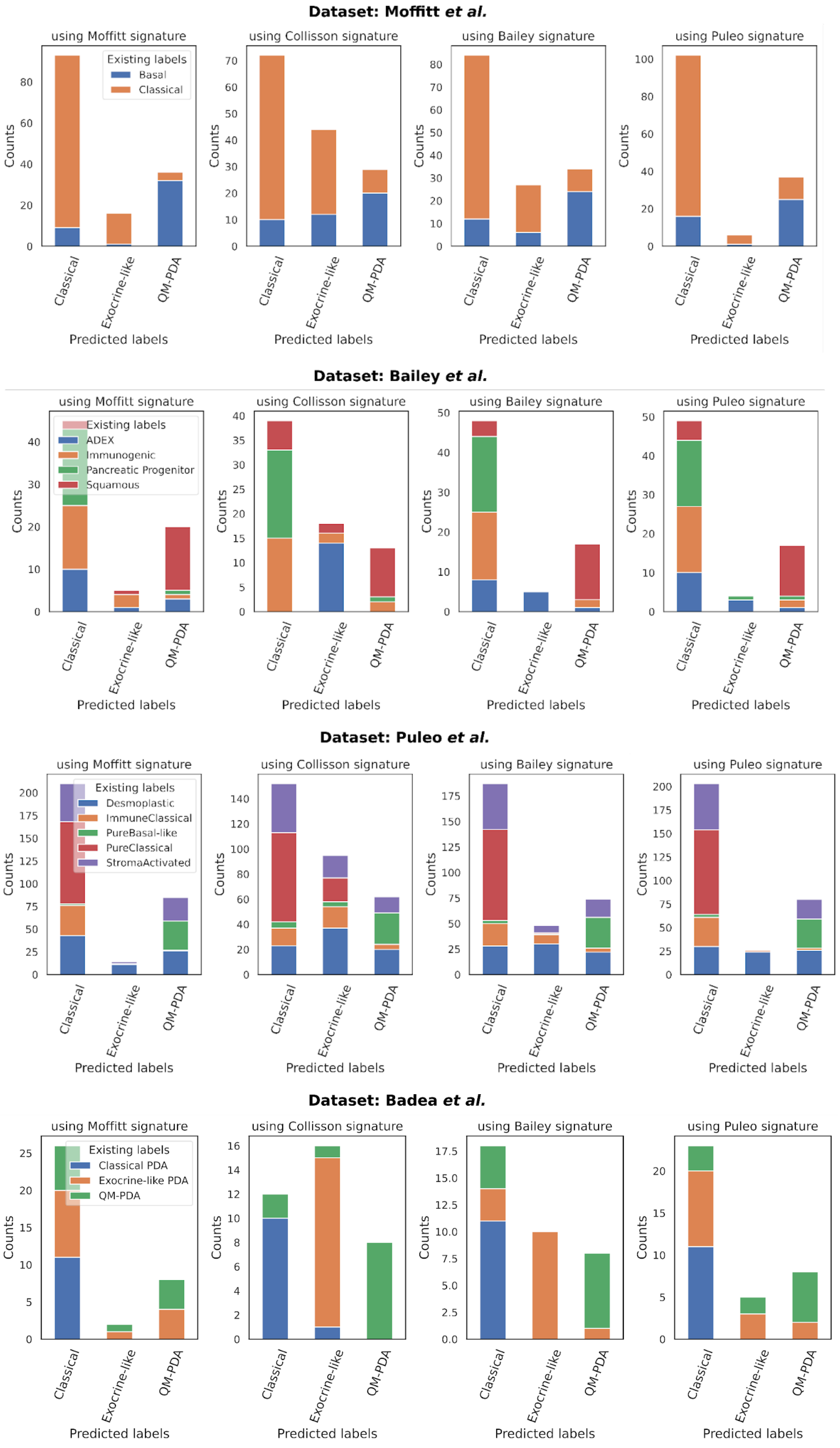

(c) Prediction of Bailey *et al.* labels

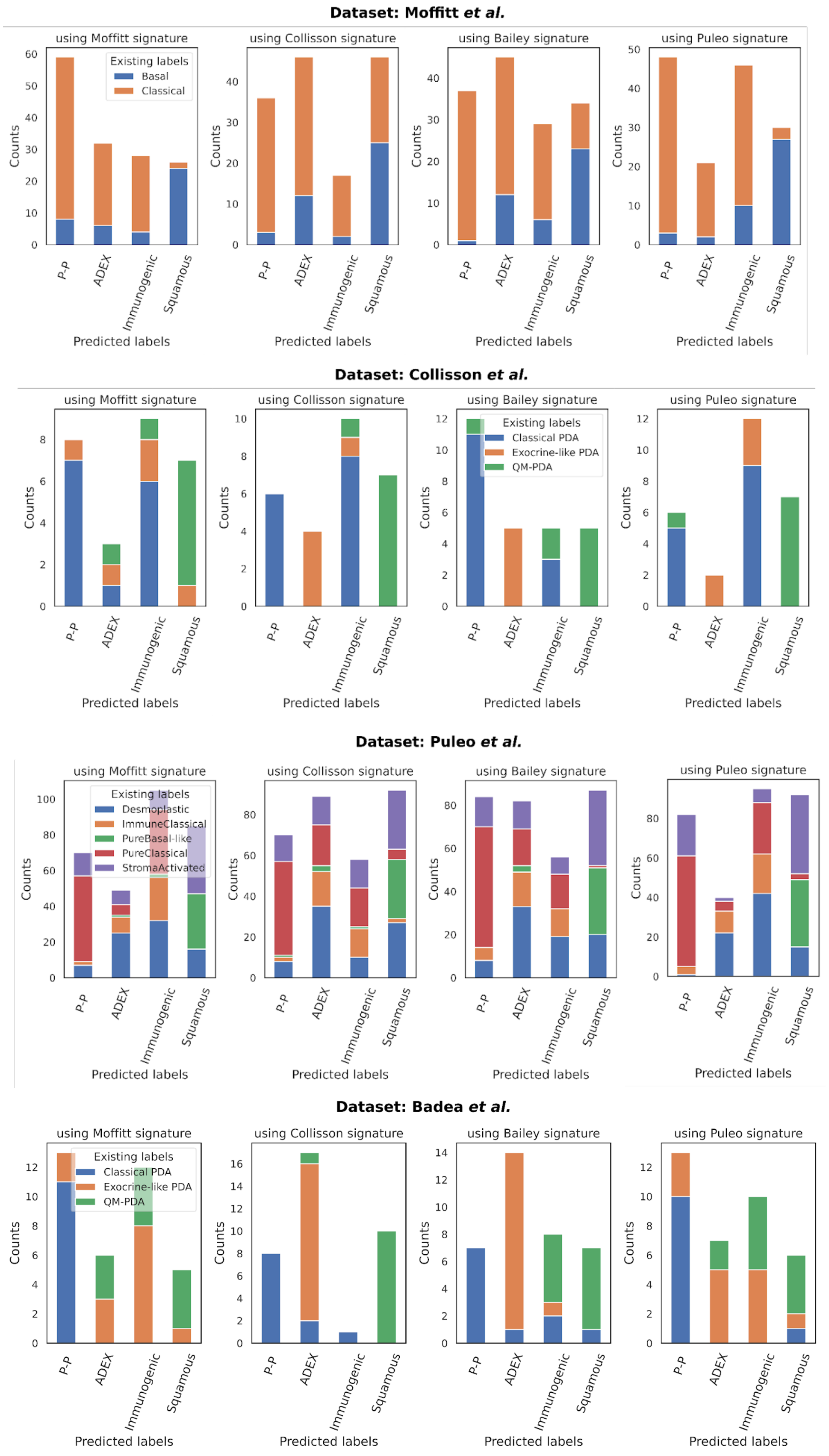

(d) Prediction of Puleo *et al.* labels

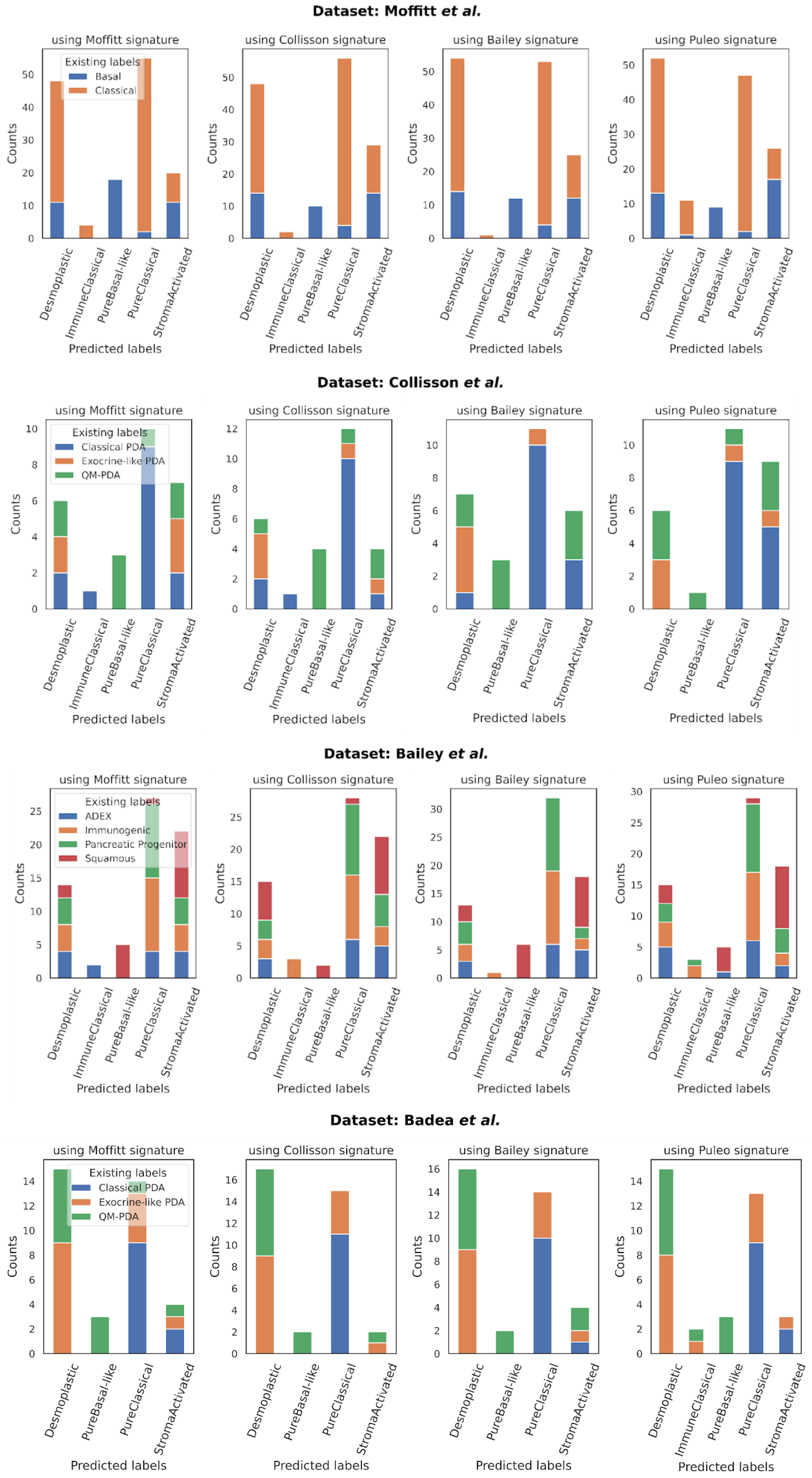

Figure S5. Comparison between predicted subtypes and existing subtypes assigned by the studies. For each classification model and for each dataset whose subtypes are known is shown the composition of the predicted subtypes, on the x-axis, with respect to the real ones. (a) Models trained on Moffitt *et al.* dataset are used to classify samples into Basal and Classical subtype using signature from Moffitt *et al.* (left), secondly Collisson *et al.* (central), then Bailey *et al.* and lastly Puleo *et al.* (right). Validation dataset considered for this comparison are the ones with existing subtype labels, excluding the same dataset used for validation. Considering one dataset at a time, we can check whether the subtype assigned to each sample through classification corresponds to the existing subtype by looking at the composition of the stacked bars in the figure. (b), (c) and (d) are the same as (a) but showing Collisson *et al.*, Bailey *et al.* and Puleo *et al.* predicted subtypes, respectively. No Immune Classical subtype was predicted for Collisson *et al.* and Badea *et al.* datasets in (d).

Figure S6

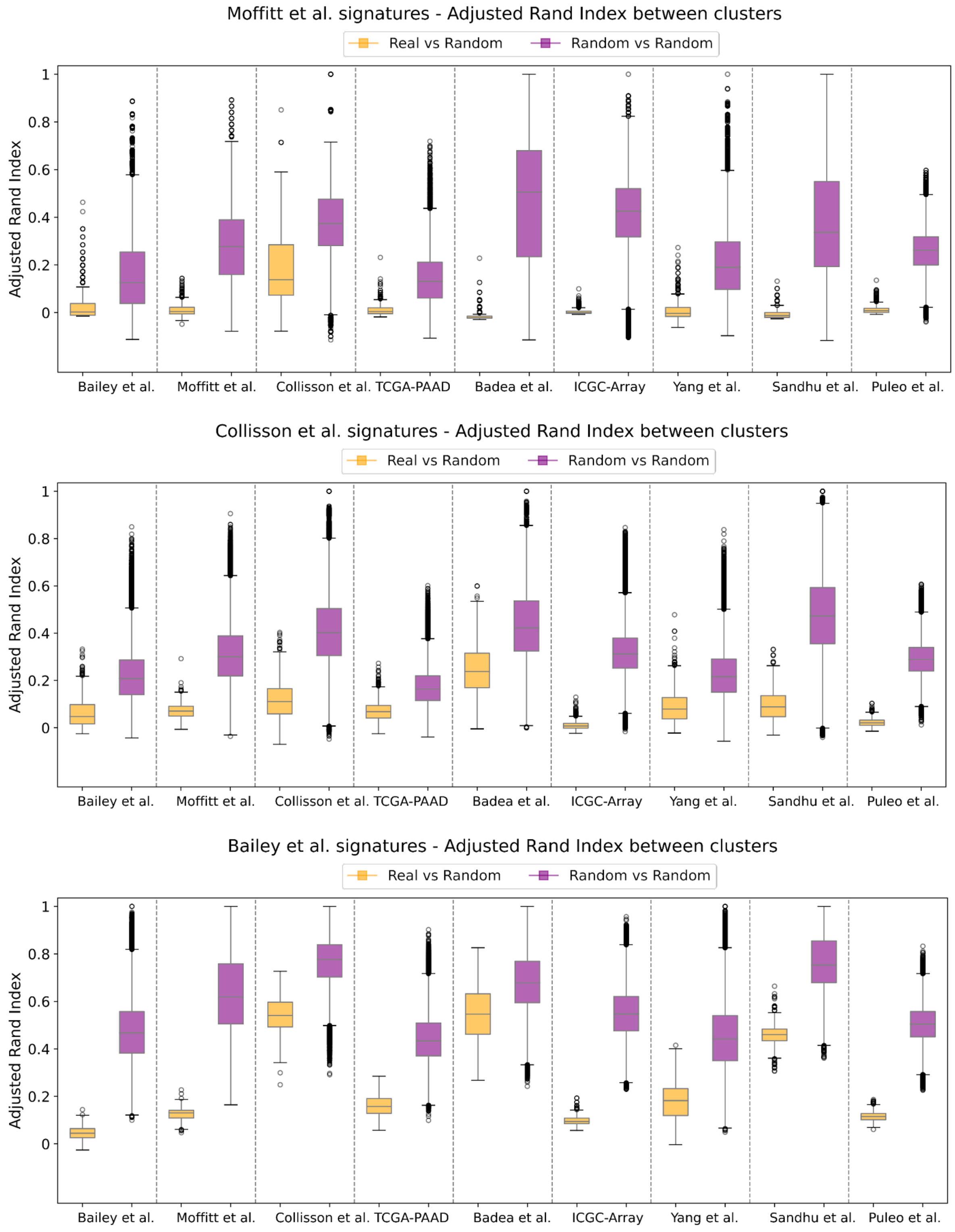

Moffitt et al. signatures - Adjusted Rand Index between clusters
Real vs Random
Random vs Random
Adjusted Rand Index
1
0.8
0.6
0.4
0.2
0
Bailey et al.
Moffitt et al.
Collisson et al.
TCGA-PAAD
Badea et al.
ICGC-Array
Yang et al.
Sandhu et al.
Puleo et al.
Collisson et al. signatures - Adjusted Rand Index between clusters
Real vs Random
Random vs Random
Adjusted Rand Index
1
0.8
0.6
0.4
0.2
0
Bailey et al.
Moffitt et al.
Collisson et al.
TCGA-PAAD
Badea et al.
ICGC-Array
Yang et al.
Sandhu et al.
Puleo et al.
Bailey et al. signatures - Adjusted Rand Index between clusters
Real vs Random
Random vs Random
Adjusted Rand Index
1
0.8
0.6
0.4
0.2
0
Bailey et al.
Moffitt et al.
Collisson et al.
TCGA-PAAD
Badea et al.
ICGC-Array
Yang et al.
Sandhu et al.
Puleo et al.

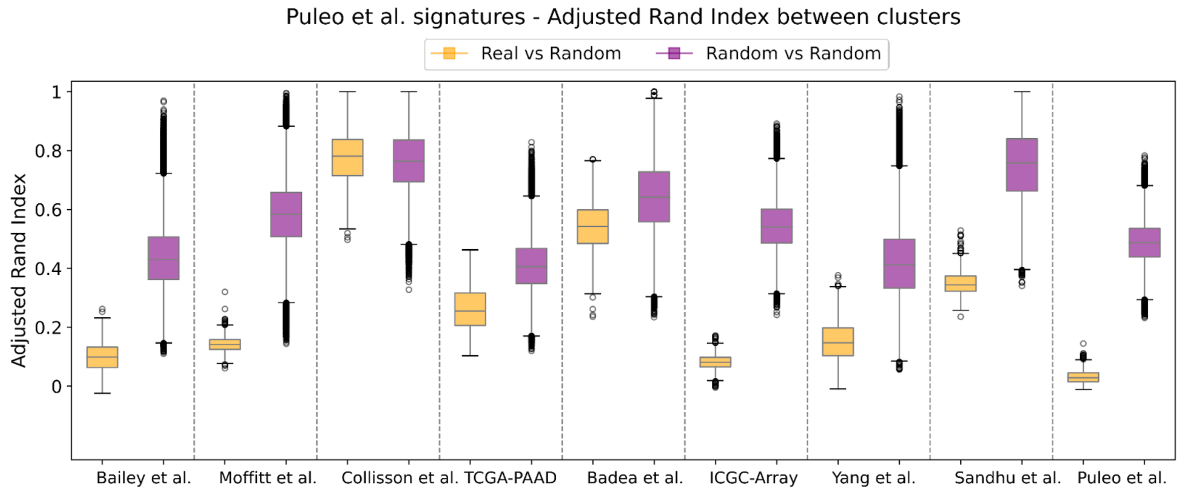

Figure S6. Adjusted Rand Index (ARI) is used for evaluating the clustering robustness of the signatures. From top to bottom: clusters derived using Moffitt *et al.*, Collisson *et al.*, Bailey *et al.* and Puleo *et al.* signatures are compared with the ones identified when employing random genes of the same size of the signature (Real vs Random). ARI is also used for a pair-wise comparison between clusters deriving from the use of random gene sets (Random vs Random).

Figure S7

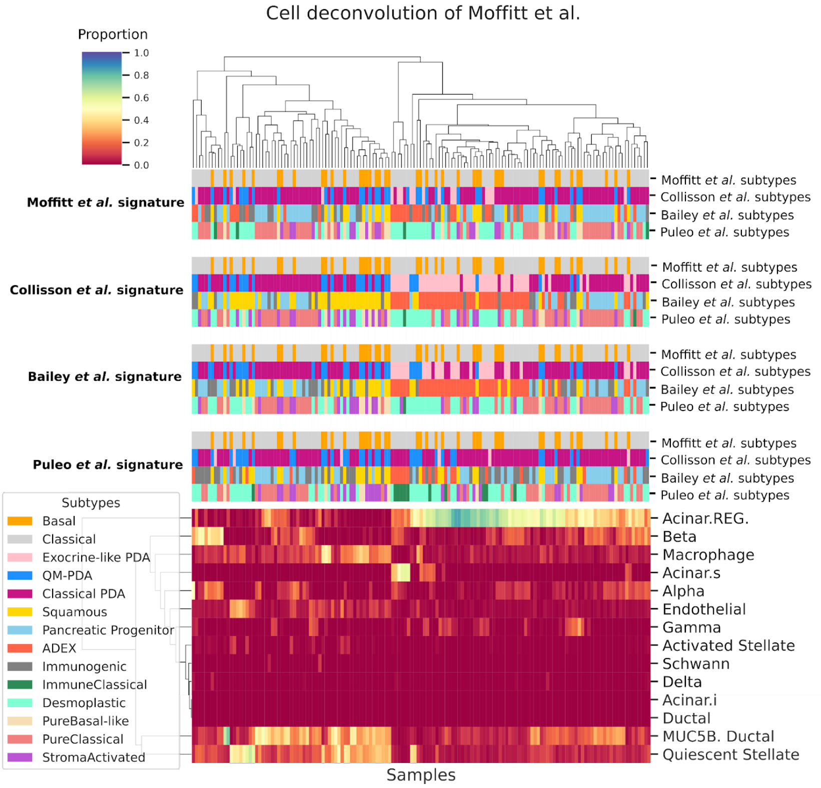
Cell deconvolution of Moffitt et al.
Proportion
1.0
0.8
0.6
0.4
0.2
0.0
Moffitt et al. signature
Collisson et al. signature
Bailey et al. signature
Puleo et al. signature
Moffitt et al. subtypes
Collisson et al. subtypes
Bailey et al. subtypes
Puleo et al. subtypes
Subtypes
Basal
Classical
Exocrine-like PDA
QM-PDA
Classical PDA
Squamous
Pancreatic Progenitor
ADEX
Immunogenic
ImmuneClassical
Desmoplastic
PureBasal-like
PureClassical
StromaActivated
Acinar.REG.
Beta
Macrophage
Acinar.s
Alpha
Endothelial
Gamma
Activated Stellate
Schwann
Delta
Acinar.i
Ductal
MUC5B. Ductal
Quiescent Stellate
Samples

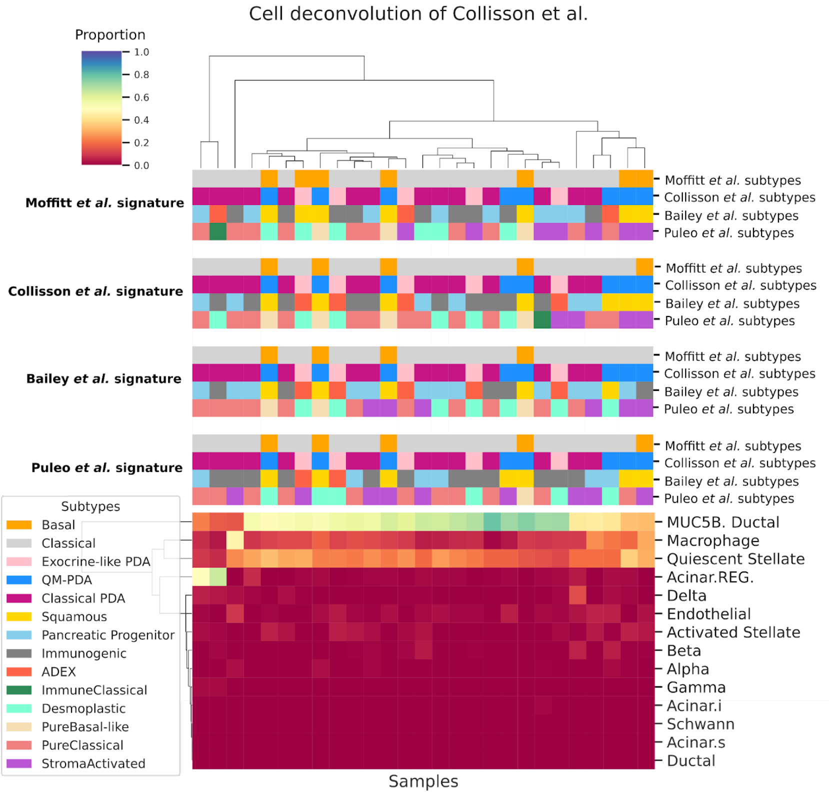
Cell deconvolution of Collisson et al.
Proportion
1.0
0.8
0.6
0.4
0.2
0.0
Moffitt et al. signature
Collisson et al. signature
Bailey et al. signature
Puleo et al. signature
Moffitt et al. subtypes
Collisson et al. subtypes
Bailey et al. subtypes
Puleo et al. subtypes
Subtypes
Basal
Classical
Exocrine-like PDA
QM-PDA
Classical PDA
Squamous
Pancreatic Progenitor
Immunogenic
ADEX
ImmuneClassical
Desmoplastic
PureBasal-like
PureClassical
StromaActivated
MUC5B. Ductal
Macrophage
Quiescent Stellate
Acinar.REG.
Delta
Endothelial
Activated Stellate
Beta
Alpha
Gamma
Acinar.i
Schwann
Acinar.s
Ductal
Samples

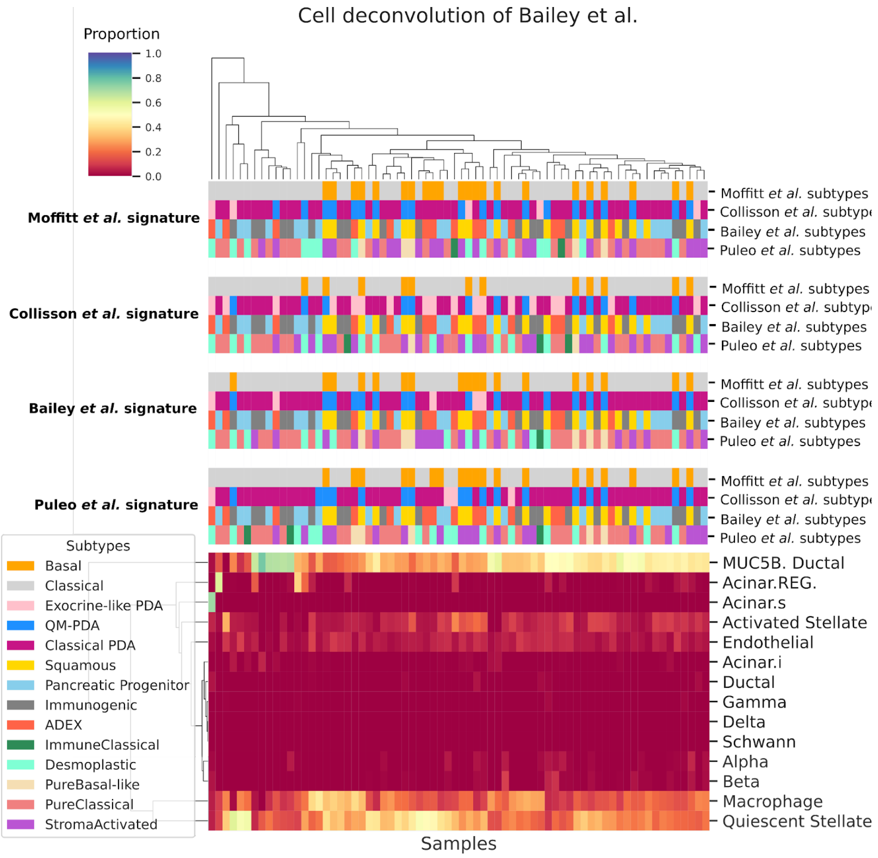

Cell deconvolution of Bailey et al.
Proportion
1.0
0.8
0.6
0.4
0.2
0.0
Moffitt et al. signature
Collisson et al. signature
Bailey et al. signature
Puleo et al. signature
Moffitt et al. subtypes
Collisson et al. subtypes
Bailey et al. subtypes
Puleo et al. subtypes
Subtypes
Basal
Classical
Exocrine-like PDA
QM-PDA
Classical PDA
Squamous
Pancreatic Progenitor
Immunogenic
ADEX
ImmuneClassical
Desmoplastic
PureBasal-like
PureClassical
StromaActivated
MUC5B. Ductal
Acinar.REG.
Acinar.s
Activated Stellate
Endothelial
Acinar.i
Ductal
Gamma
Delta
Schwann
Alpha
Beta
Macrophage
Quiescent Stellate
Samples

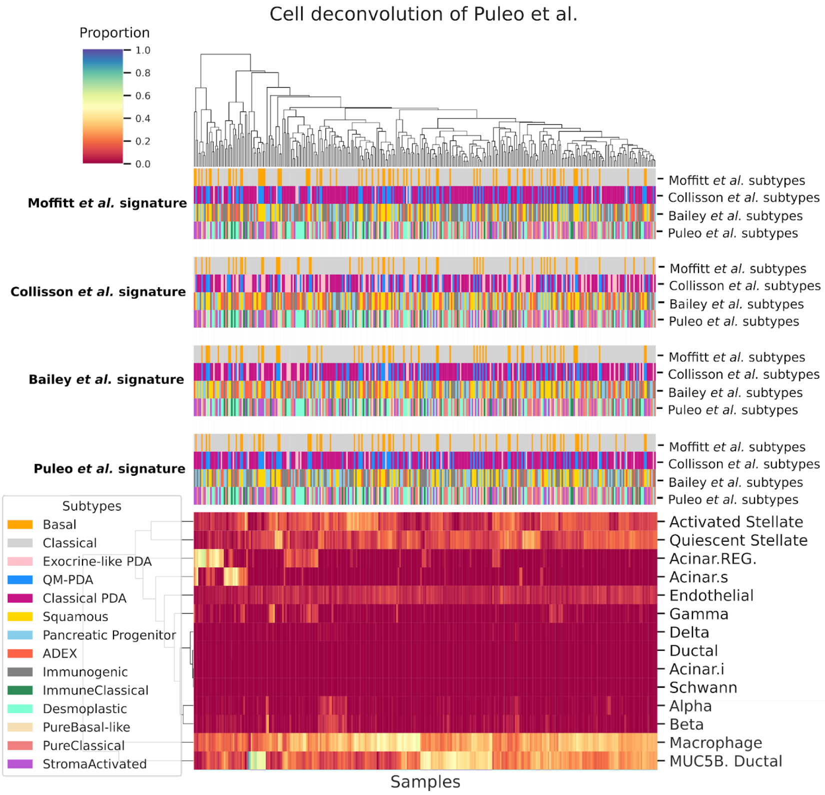

Cell deconvolution of Puleo et al.
Proportion
1.0
0.8
0.6
0.4
0.2
0.0
Moffitt et al. signature
Collisson et al. signature
Bailey et al. signature
Puleo et al. signature
Moffitt et al. subtypes
Collisson et al. subtypes
Bailey et al. subtypes
Puleo et al. subtypes
Subtypes
Basal
Classical
Exocrine-like PDA
QM-PDA
Classical PDA
Squamous
Pancreatic Progenitor
ADEX
Immunogenic
ImmuneClassical
Desmoplastic
PureBasal-like
PureClassical
StromaActivated
Activated Stellate
Quiescent Stellate
Acinar.REG.
Acinar.s
Endothelial
Gamma
Delta
Ductal
Acinar.i
Schwann
Alpha
Beta
Macrophage
MUC5B. Ductal
Samples

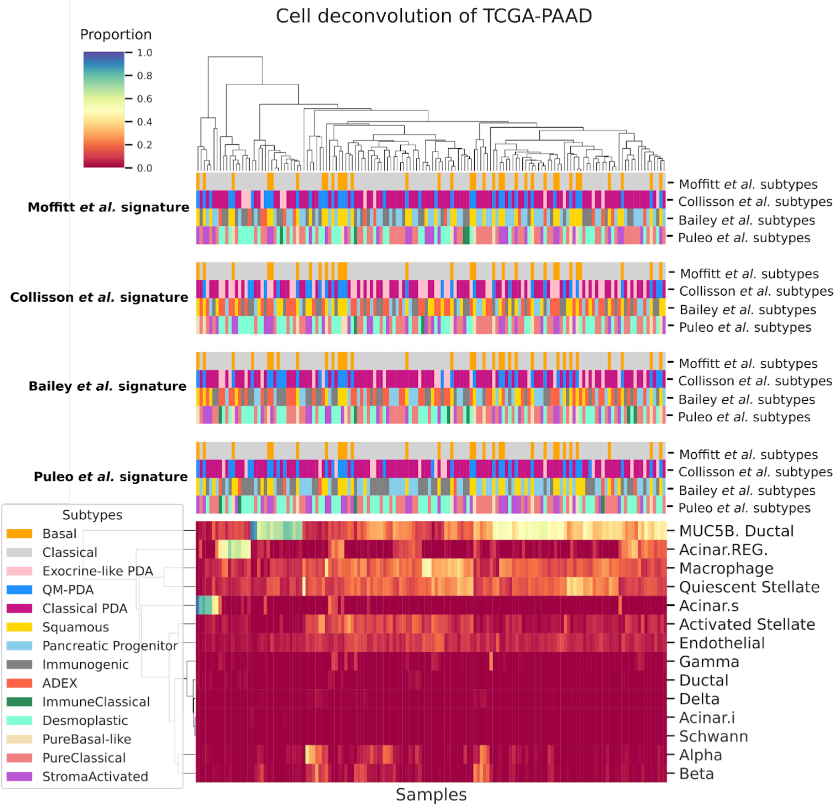
Cell deconvolution of TCGA-PAAD
Proportion
1.0
0.8
0.6
0.4
0.2
0.0
Moffitt et al. signature
Collisson et al. signature
Bailey et al. signature
Puleo et al. signature
Moffitt et al. subtypes
Collisson et al. subtypes
Bailey et al. subtypes
Puleo et al. subtypes
Subtypes
Basal
Classical
Exocrine-like PDA
QM-PDA
Classical PDA
Squamous
Pancreatic Progenitor
Immunogenic
ADEX
ImmuneClassical
Desmoplastic
PureBasal-like
PureClassical
StromaActivated
MUC5B. Ductal
Acinar.REG.
Macrophage
Quiescent Stellate
Acinar.s
Activated Stellate
Endothelial
Gamma
Ductal
Delta
Acinar.i
Schwann
Alpha
Beta
Samples

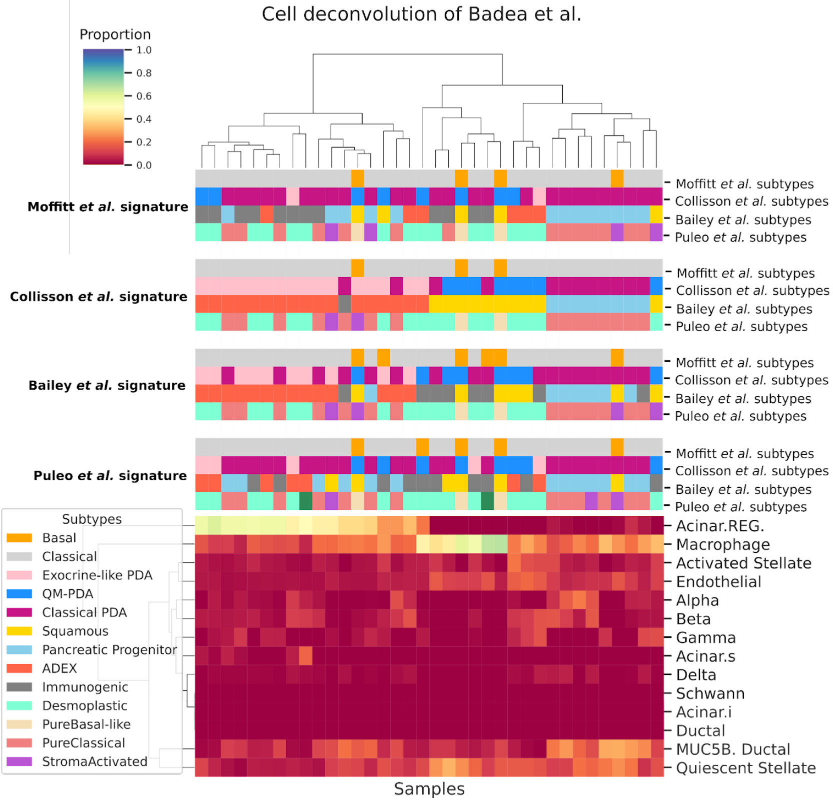
Cell deconvolution of Badea et al.
Proportion
1.0
0.8
0.6
0.4
0.2
0.0
Moffitt et al. signature
Collisson et al. signature
Bailey et al. signature
Puleo et al. signature
Moffitt et al. subtypes
Collisson et al. subtypes
Bailey et al. subtypes
Puleo et al. subtypes
Subtypes
Basal
Classical
Exocrine-like PDA
QM-PDA
Classical PDA
Squamous
Pancreatic Progenitor
ADEX
Immunogenic
Desmoplastic
PureBasal-like
PureClassical
StromaActivated
Acinar.REG.
Macrophage
Activated Stellate
Endothelial
Alpha
Beta
Gamma
Acinar.s
Delta
Schwann
Acinar.i
Ductal
MUC5B. Ductal
Quiescent Stellate
Samples

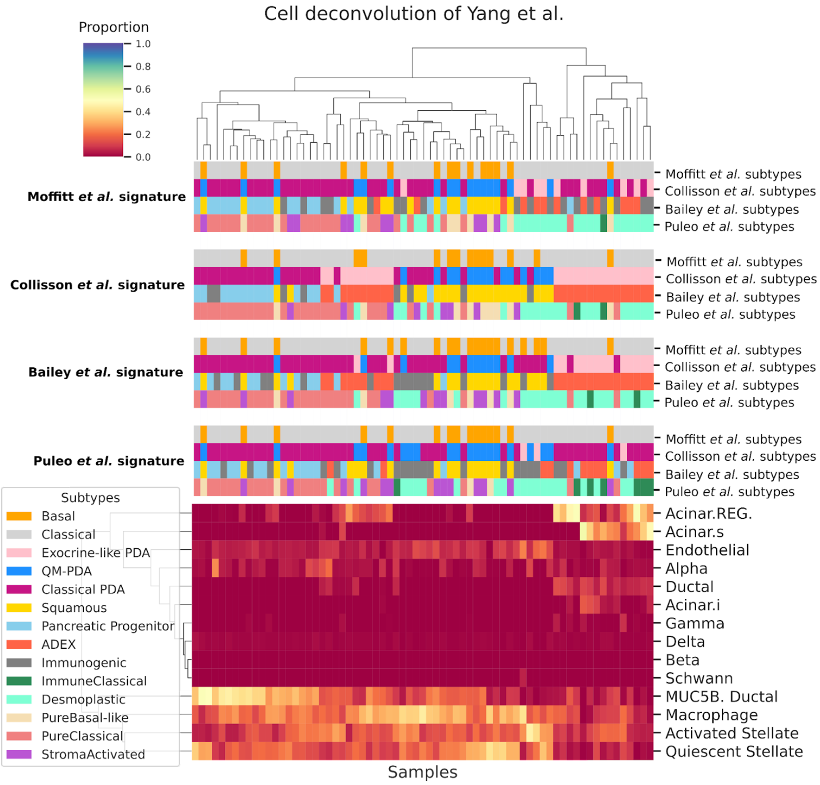
Cell deconvolution of Yang et al.
Proportion
1.0
0.8
0.6
0.4
0.2
0.0
Moffitt et al. signature
Collisson et al. signature
Bailey et al. signature
Puleo et al. signature
Moffitt et al. subtypes
Collisson et al. subtypes
Bailey et al. subtypes
Puleo et al. subtypes
Subtypes
Basal
Classical
Exocrine-like PDA
QM-PDA
Classical PDA
Squamous
Pancreatic Progenitor
ADEX
Immunogenic
ImmuneClassical
Desmoplastic
PureBasal-like
PureClassical
StromaActivated
Acinar.REG.
Acinar.s
Endothelial
Alpha
Ductal
Acinar.i
Gamma
Delta
Beta
Schwann
MUC5B. Ductal
Macrophage
Activated Stellate
Quiescent Stellate
Samples

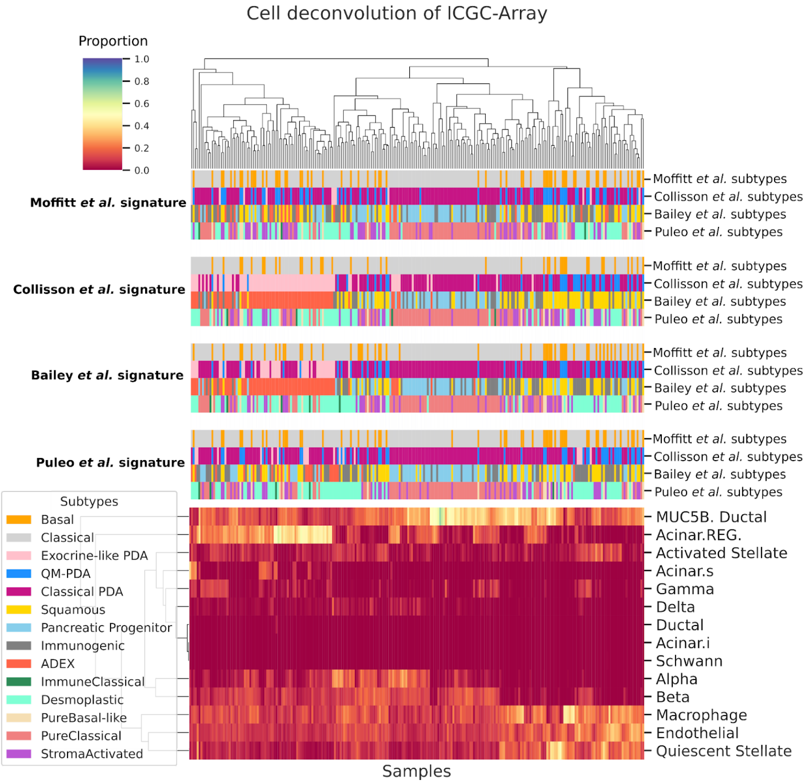
Cell deconvolution of ICGC-Array
Proportion
1.0
0.8
0.6
0.4
0.2
0.0
Moffitt et al. signature
Collisson et al. signature
Bailey et al. signature
Puleo et al. signature
Moffitt et al. subtypes
Collisson et al. subtypes
Bailey et al. subtypes
Puleo et al. subtypes
Subtypes
Basal
Classical
Exocrine-like PDA
QM-PDA
Classical PDA
Squamous
Pancreatic Progenitor
Immunogenic
ADEX
ImmuneClassical
Desmoplastic
PureBasal-like
PureClassical
StromaActivated
MUC5B. Ductal
Acinar.REG.
Activated Stellate
Acinar.s
Gamma
Delta
Ductal
Acinar.i
Schwann
Alpha
Beta
Macrophage
Endothelial
Quiescent Stellate
Samples

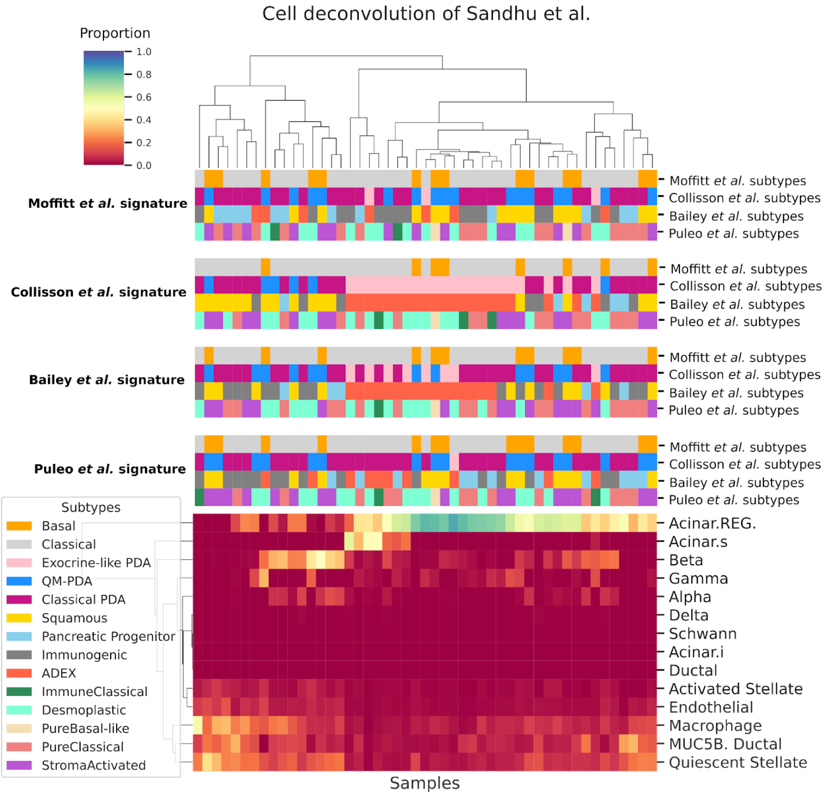

Figure S7. Hierarchical clustering of the inferred proportion of pancreatic cell types (on the rows) enriched in the nine cohorts. For each dataset are shown the classes predicted by the sixteen predictor models.

Figure S8

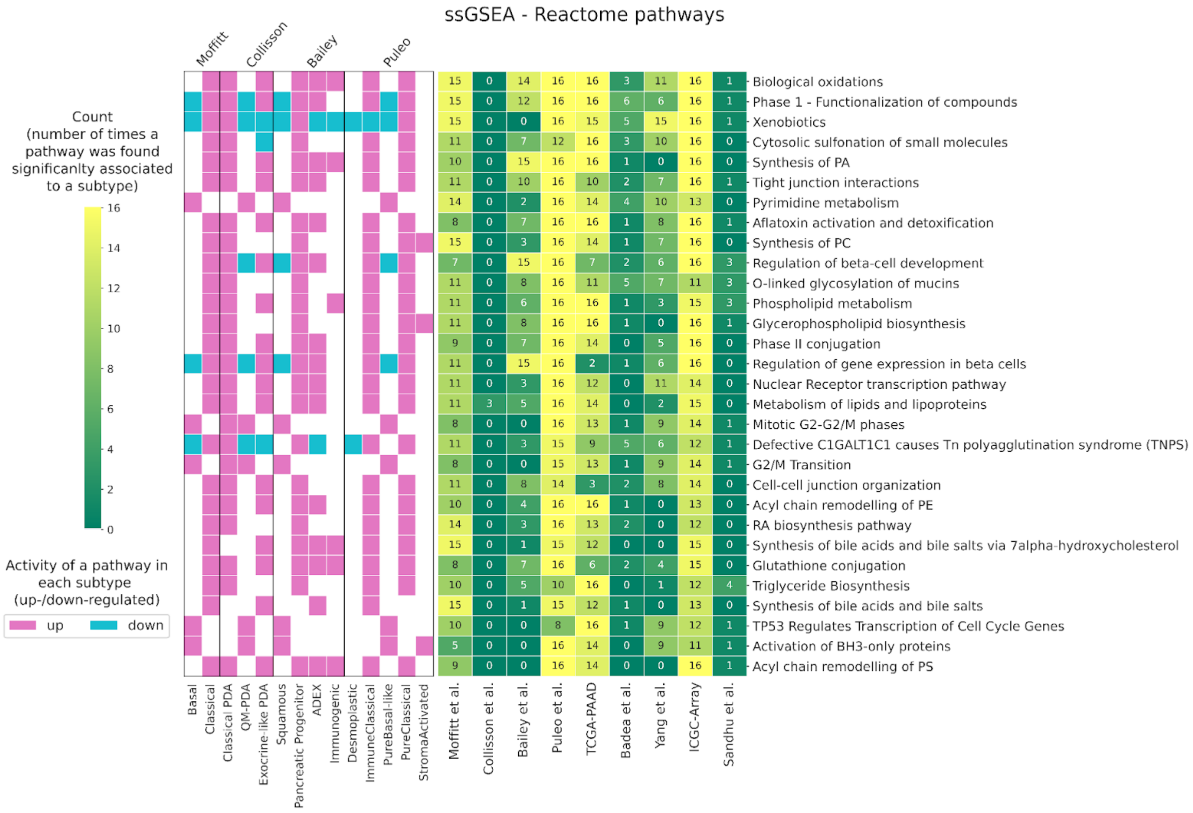
ssGSEA - Reactome pathways
Moffitt
Collisson
Bailey
Puleo
Count
(number of times a pathway was found significanlty associated to a subtype)
16
14
12
10
8
6
4
2
0
Activity of a pathway in each subtype (up-/down-regulated)
up
down
Basal
Classical
Classical PDA
QM-PDA
Exocrine-like PDA
Squamous
Pancreatic Progenitor
ADEX
Immunogenic
Desmoplastic
ImmuneClassical
PureBasal-like
PureClassical
StromaActivated
Moffitt et al.
Collisson et al.
Bailey et al.
Puleo et al.
TCGA-PAAD
Badea et al.
Yang et al.
ICGC-Array
Sandhu et al.
15 0 14 16 16 3 11 16 1 Biological oxidations
15 0 12 16 16 6 6 16 1 Phase 1 - Functionalization of compounds
15 0 0 16 15 5 15 16 1 Xenobiotics
11 0 7 12 16 3 10 16 0 Cytosolic sulfonation of small molecules
10 0 15 16 16 1 0 16 0 Synthesis of PA
11 0 10 16 10 2 7 16 1 Tight junction interactions
14 0 2 16 14 4 10 13 0 Pyrimidine metabolism
8 0 7 16 16 1 8 16 1 Aflatoxin activation and detoxification
15 0 3 16 14 1 7 16 0 Synthesis of PC
7 0 15 16 7 2 6 16 3 Regulation of beta-cell development
11 0 8 16 11 5 7 11 3 O-linked glycosylation of mucins
11 0 6 16 16 1 3 15 3 Phospholipid metabolism
11 0 8 16 16 1 0 16 1 Glycerophospholipid biosynthesis
9 0 7 16 14 0 5 16 0 Phase II conjugation
11 0 15 16 2 1 6 16 0 Regulation of gene expression in beta cells
11 0 3 16 12 0 11 14 0 Nuclear Receptor transcription pathway
11 3 5 16 14 0 2 15 0 Metabolism of lipids and lipoproteins
8 0 0 16 13 1 9 14 1 Mitotic G2-G2/M phases
11 0 3 15 9 5 6 12 1 Defective C1GALT1C1 causes Tn polyagglutination syndrome (TNPS)
8 0 0 15 13 1 9 14 1 G2/M Transition
11 0 8 14 3 2 8 14 0 Cell-cell junction organization
10 0 4 16 16 1 0 13 0 Acyl chain remodelling of PE
14 0 3 16 13 2 0 12 0 RA biosynthesis pathway
15 0 1 15 12 0 0 15 0 Synthesis of bile acids and bile salts via 7alpha-hydroxycholesterol
8 0 7 16 6 2 4 15 0 Glutathione conjugation
10 0 5 10 16 0 1 12 4 Triglyceride Biosynthesis
15 0 1 15 12 1 0 13 0 Synthesis of bile acids and bile salts
10 0 0 8 16 1 9 12 1 TP53 Regulates Transcription of Cell Cycle Genes
5 0 0 16 14 0 9 11 1 Activation of BH3-only proteins
9 0 0 16 14 0 0 16 1 Acyl chain remodelling of PS

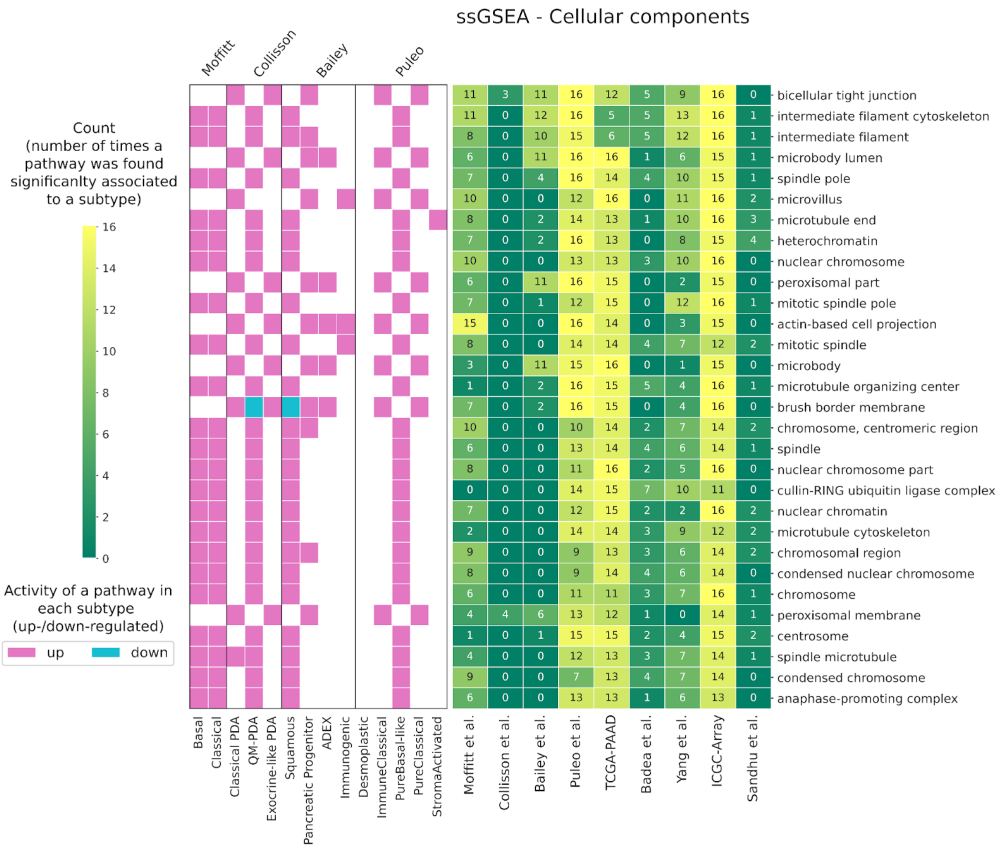
ssGSEA - Cellular components
Moffitt
Collisson
Bailey
Puleo
Count
(number of times a
pathway was found
significanlty associated
to a subtype)
16
14
12
10
8
6
4
2
0
Activity of a pathway in
each subtype
(up-/down-regulated)
up
down
bicellular tight junction
intermediate filament cytoskeleton
intermediate filament
microbody lumen
spindle pole
microvillus
microtubule end
heterochromatin
nuclear chromosome
peroxisomal part
mitotic spindle pole
actin-based cell projection
mitotic spindle
microbody
microtubule organizing center
brush border membrane
chromosome, centromeric region
spindle
nuclear chromosome part
cullin-RING ubiquitin ligase complex
nuclear chromatin
microtubule cytoskeleton
chromosomal region
condensed nuclear chromosome
chromosome
peroxisomal membrane
centrosome
spindle microtubule
condensed chromosome
anaphase-promoting complex
Basal
Classical
Classical PDA
QM-PDA
Exocrine-like PDA
Squamous
Pancreatic Progenitor
ADEX
Immunogenic
Desmoplastic
ImmuneClassical
PureBasal-like
PureClassical
StromaActivated
Moffitt et al.
Collisson et al.
Bailey et al.
Puleo et al.
TCGA-PAAD
Badea et al.
Yang et al.
ICGC-Array
Sandhu et al.

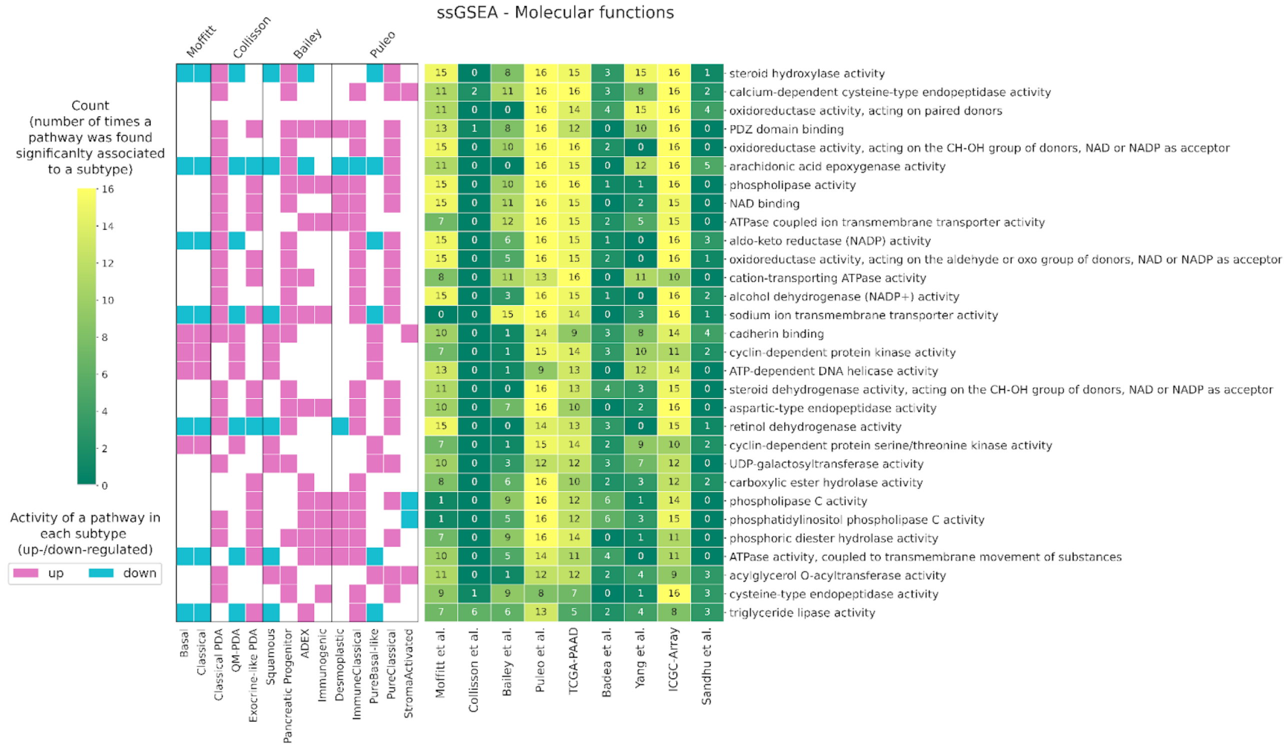
ssGSEA - Molecular functions
Moffitt
Collisson
Bailey
Puleo
Count
(number of times a
pathway was found
significanlty associated
to a subtype)
16
14
12
10
8
6
4
2
0
Activity of a pathway in
each subtype
(up-/down-regulated)
up
down
steroid hydroxylase activity
calcium-dependent cysteine-type endopeptidase activity
oxidoreductase activity, acting on paired donors
PDZ domain binding
oxidoreductase activity, acting on the CH-OH group of donors, NAD or NADP as acceptor
arachidonic acid epoxygenase activity
phospholipase activity
NAD binding
ATPase coupled ion transmembrane transporter activity
aldo-keto reductase (NADP) activity
oxidoreductase activity, acting on the aldehyde or oxo group of donors, NAD or NADP as acceptor
cation-transporting ATPase activity
alcohol dehydrogenase (NADP+) activity
sodium ion transmembrane transporter activity
cadherin binding
cyclin-dependent protein kinase activity
ATP-dependent DNA helicase activity
steroid dehydrogenase activity, acting on the CH-OH group of donors, NAD or NADP as acceptor
aspartic-type endopeptidase activity
retinol dehydrogenase activity
cyclin-dependent protein serine/threonine kinase activity
UDP-galactosyltransferase activity
carboxylic ester hydrolase activity
phospholipase C activity
phosphatidylinositol phospholipase C activity
phosphoric diester hydrolase activity
ATPase activity, coupled to transmembrane movement of substances
acylglycerol O-acyltransferase activity
cysteine-type endopeptidase activity
triglyceride lipase activity
Basal
Classical
Classical PDA
QM-PDA
Exocrine-like PDA
Squamous
Pancreatic Progenitor
ADEX
Immunogenic
Desmoplastic
ImmuneClassical
PureBasal-like
PureClassical
StromaActivated
Moffitt et al.
Collisson et al.
Bailey et al.
Puleo et al.
TCGA-PAAD
Badea et al.
Yang et al.
ICGC-Array
Sandhu et al.

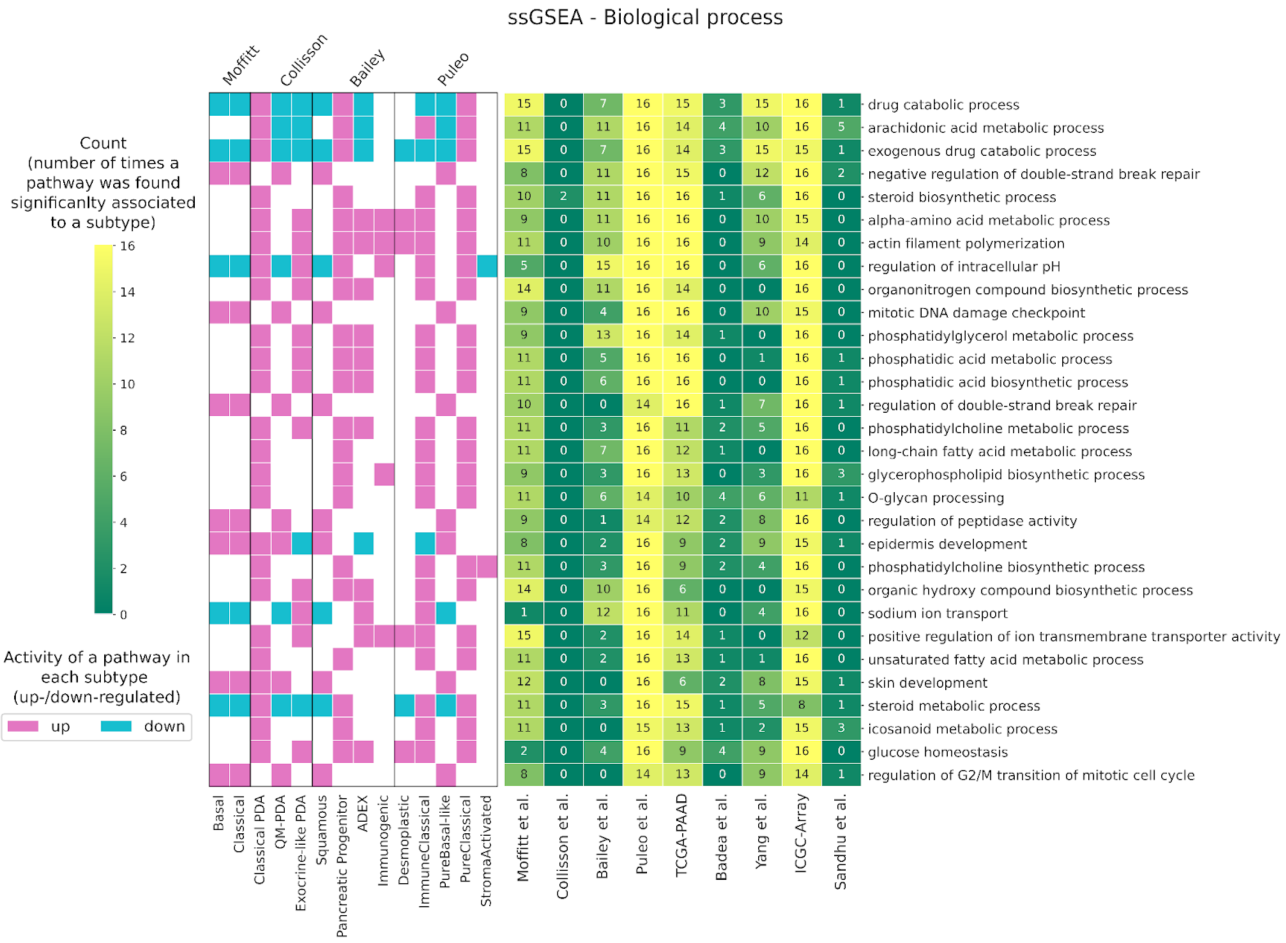

Figure S8. ssGSEA of the nine cohorts. Enrichment scores of terms are compared between predicted subtypes and across cohorts and the top 30 most associated are counted and shown. For each term we know whether it was found up or down regulated and in which subtype.
